# Detecting and characterizing restricted diffusion with accessible magnetic field gradients

**DOI:** 10.1093/pnasnexus/pgag293

**Published:** 2026-09-05

**Authors:** Alfredo Ordinola, Magnus Herberthson, Walker Scot Jackson, Evren Özarslan

**Affiliations:** Department of Biomedical Engineering (IMT), Linköping University, Linköping S-58185, Sweden; Department of Mathematics (MAI), Linköping University, Linköping S-58330, Sweden; Department of Biomedical and Clinical Sciences (BKV), Linköping University, Linköping S-58185, Sweden; Luxembourg Centre for Systems Biomedicine, University of Luxembourg, L-4367 Belvaux, Luxembourg; Department of Biomedical Engineering (IMT), Linköping University, Linköping S-58185, Sweden; Center for Medical Image Science and Visualization (CMIV), Linköping University, Linköping, Sweden

**Keywords:** diffusion MRI, restricted diffusion, microstructure, gradient waveform, benchtop MRI

## Abstract

For over 50 years, diffusion magnetic resonance has been employed in numerous studies on characterizing chemical compounds, porous media, and biological tissues. These studies have almost exclusively employed the Stejskal–Tanner (ST) pulse sequence, which is sensitive to the ensemble average propagator (EAP). EAP is a compromised version of the true diffusion propagator—a quantity that fully describes the diffusion process in any system and holds exquisite information that can be used to characterize complex samples. Recently, a new diffusion sensitization method was proposed, here referred to as PROP, and was shown to be capable of acquiring the true diffusion propagator in closed pores. However, its adequate use requires a large number of measurements and (consequently) a long acquisition time. Thus, its translation to, e.g. the clinical field, is challenging. Here, we propose a reduced PROP pulse sequence (rPROP) which offers various advantages over the ST sequence. rPROP excels at isolating restricted diffusion thanks to its inherently high diffusion-weighting, yielding an efficient free diffusion filter. We employ rPROP to study different samples, and compare its performance to ST measurements in terms of estimation accuracy and gradient amplitude requirements. Furthermore, we study the capability of rPROP to detect anisotropic diffusion in samples which predominantly feature isotropic diffusion. Finally, we present a method capable of acquiring the mean position distribution (analogous to the EAP attainable via ST data) of particles via MR measurements. We highlight that all signals and images presented in this work were acquired using a benchtop MRI scanner.

Significance Statement This work introduces a novel diffusion method, rPROP, that improves upon the widely used Stejskal–Tanner (ST) experiment by more effectively isolating restricted diffusion in complex media. The rPROP technique alleviates the hardware requirements of the ST method without increasing the demand on the number of acquisitions. We demonstrate rPROP’s advantages in detecting subtle diffusion features, including anisotropy in predominantly isotropic samples, using a benchtop MRI system. These findings pave the way for more informative and accessible diffusion imaging across a range of applications—from porous materials to biomedical contexts—while addressing key limitations of existing techniques.

## Introduction

Microstructure of complex media has been studied employing various magnetic resonance (MR) modalities. One such modality is diffusion magnetic resonance (dMR), which quantifies the ensemble behavior of spin-bearing particles’ trajectories influenced by the microscopic features of the environment. Ever since its introduction in 1965, the Stejskal–Tanner (ST) pulse sequence (1) has dominated such dMR studies, resulting in techniques such as diffusion weighted imaging (DWI) and diffusion tensor imaging (DTI) (2). The power of these techniques is due to their sensitivity to various features of the diffusion propagator. The diffusion propagator is a conditional probability distribution indicating the likelihood of particles moving from their initial to final positions over certain time. As such, it has one temporal and six spatial variables as its arguments. The propagator completely describes diffusion-related processes in any system (3), capturing the effect of, for example, multiple (free or restricted) compartments’ size, diffusivities, and arrangement. Throughout the years, different methods aiming at estimating the diffusion propagator, or quantities related to it, have been proposed (1, 4–6). For example, with data from the aforementioned ST sequence, one is capable of estimating the ensemble average propagator (EAP), which is the integral of the true propagator over the initial positions of the particles, hence upon a change-of-variables provides a distribution of net displacements irrespective of the initial positions (7). Another quantity that has been recovered via dMR is the steady state distribution of the particles, which is just the diffusion propagator at long diffusion times. This information was used by Laun et al. to determine the boundaries restricting the diffusion process giving rise to diffusion pore space imaging (PSI) (6, 8).

**Figure 1:**
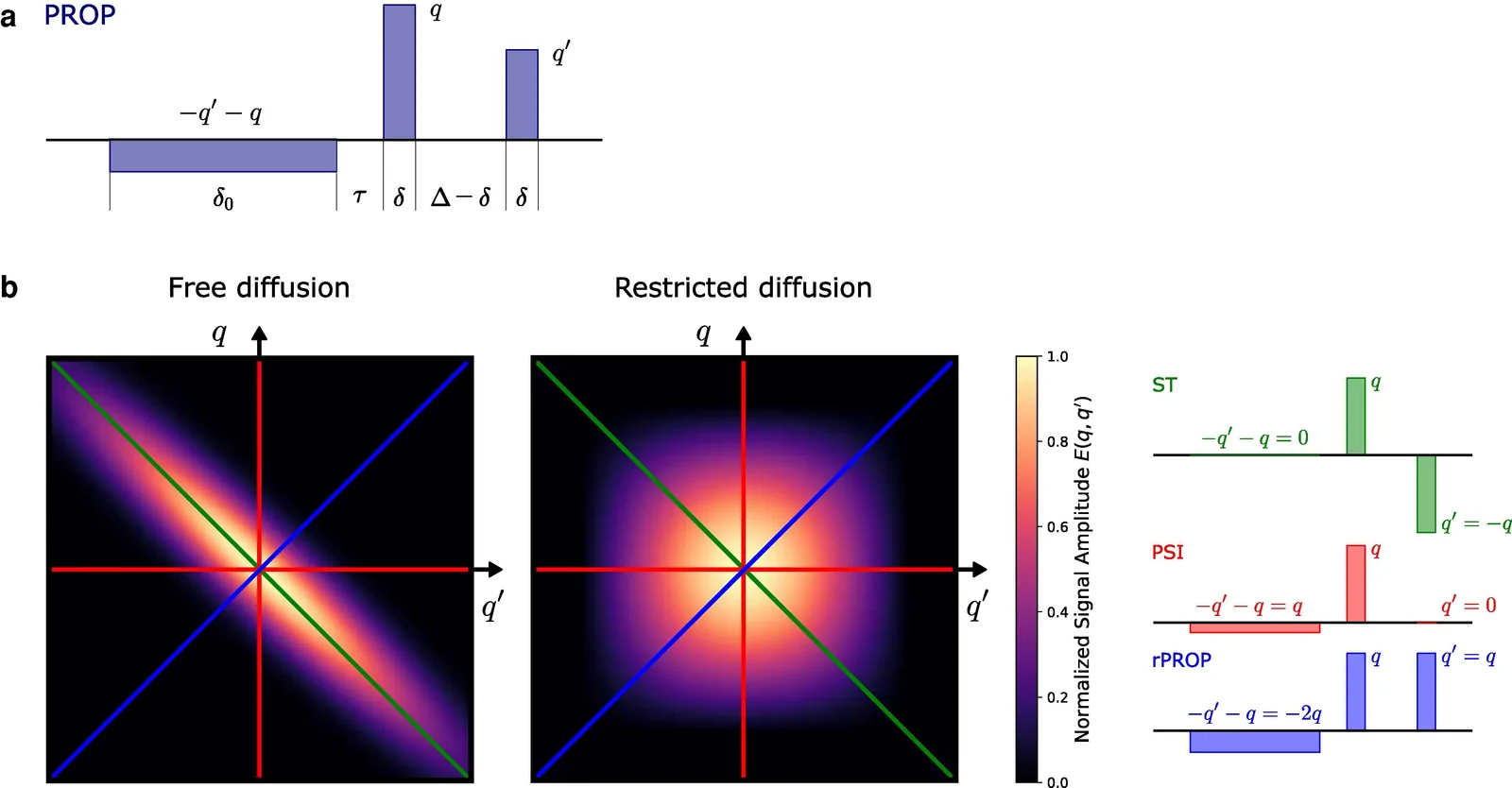
a) Propagator-sensitive diffusion encoding gradient waveform (PROP (10)). The effective waveform is shown here, i.e. the effects of RF pulses are not included. b) Data simulation for free and restricted diffusion depicting the signal amplitude for different combinations of *q* and $q^{\prime }$ in a 1D realization of the sequence in (a), and effective waveforms obtained for special cases of the waveform. The traditional ST acquisition (1) is obtained for $q^{\prime }=-q$ (green), while Laun et al.’s waveform (6) is available with q=0 or $q^{\prime }=0$ (red). In this work, we introduce the case where $q^{\prime }=q$ (blue), which we refer to as rPROP.

Recently, a new gradient waveform (here referred to as PROP and illustrated in Fig. 1a) was shown to be capable of reconstructing the true diffusion propagator in fully restricted compartments (9, 10), and for free diffusion (11). This technique employs a large number of acquisitions to reconstruct the diffusion propagator since a $qq^{\prime }$ plane needs to be sampled, where *q* and $q^{\prime }$ are the time integrals of the narrower gradient pulses multiplied by the gyromagnetic ratio of the spin-bearing nuclei. As can be seen in Fig. 1b, the ST and Laun et al.’s sequences (1, 6) are special cases of the PROP sequence, sampling the $qq^{\prime }$ plane along the green ($q^{\prime }=-q$) and red (q=0 or $q^{\prime }=0$) lines, respectively. In this work, we propose employing another subset of data indicated by the blue ($q^{\prime }=q$) line, yielding a special sequence we refer to as rPROP. Thus, the number of samples required would match that employed for performing either of the aforementioned sequences.

In the backgrounds of the $qq^{\prime }$ planes in Fig. 1b, we show the simulated signals for free and restricted diffusion processes. The free diffusion signal is heavily suppressed in rPROP for the same *q*-value (or gradient amplitude) when compared to ST (left). This is due to the much higher diffusion weighting (commonly quantified by the *b*-value (12)) obtained with rPROP when employing the same value of *q*, and therefore maximum gradient amplitude, as ST; more details are presented in the Theory section. On the other hand, the simulated restricted diffusion signal (in the long time regime) is maintained with rPROP to approximately the same degree as with ST (right). Therefore, we employ rPROP as a free diffusion filter (i.e. for isolating the part of the signal corresponding to restricted diffusion) to study different media, such as oil–water emulsions and biological tissue throughout this work. Comparisons with additional gradient waveforms are included in the Supplementary material.

In this study, we first show rPROP’s filtering capabilities and that signals acquired via this measurement can be modeled with a fully restricted model at low-to-mid *q*-values, reducing the demand on hardware for such a study. We illustrate this filtering effect in signals acquired from an oil–water emulsion (dairy cream [DC]).

Furthermore, since diffusion anisotropy typically arises from restricted diffusion (13), we use rPROP to detect anisotropy in samples with an overwhelming presence of isotropic diffusion. This could be important for applications such as fiber-tract mapping in the brain, where the smallest trace of anisotropy could be indicative of an important connection. We illustrate this application in spectroscopy-like data from a celery sample, which features an extremely low contribution of regions with anisotropic diffusion.

Finally, we perform a spectral analysis of a 1D diffusion encoding measurement to reconstruct the distribution of the mean values of the particles’ positions at two time points. This mean position distribution (MPD) is compared with the EAP available via ST, both reconstructed from data corresponding to a DC sample, and different areas of an excised spinal cord (SC) sample.

## Results

### Detecting restricted diffusion in DC

**Figure 2:**
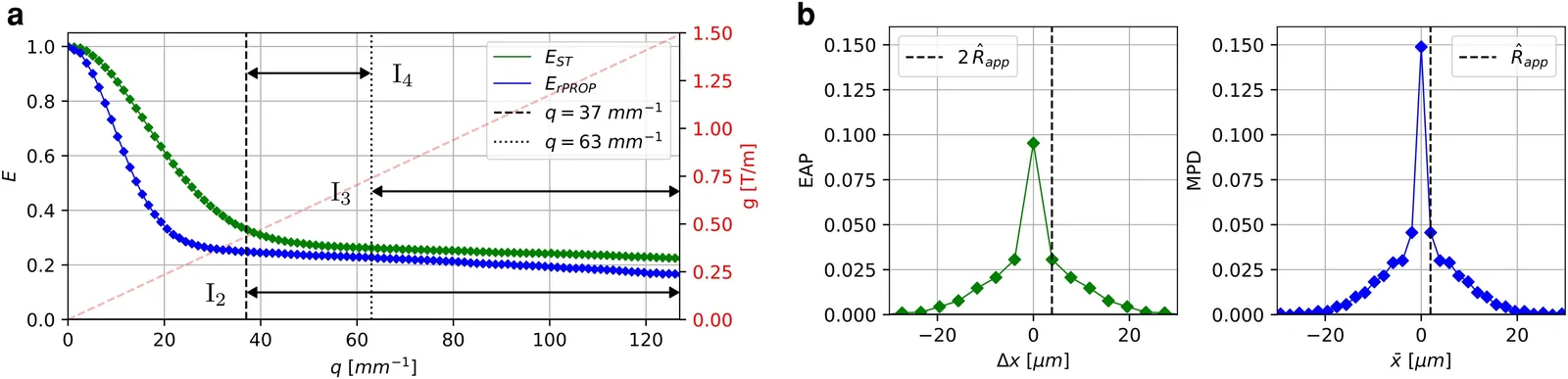
Results of experiments on the DC sample. a) Signals acquired for the ST (in green) and rPROP (in blue) versions of the PROP sequence (i.e. $q^{\prime }=-q$ and $q^{\prime }=q$, respectively). In dashed and dotted lines are the limits of the *q*-ranges defined in Estimation of the system’s parameters in experimental data section. The red-dashed line corresponds to the gradient amplitude required (right-hand *y*-axis) to achieve the corresponding *q*-value for either sequence. b) Estimated EAP (left) and MPD (right) obtained from the full range of *q*-values.

In order to assess the capability of rPROP to isolate the contribution of a restricted compartment in MR signals, data from a DC sample was acquired with both ST and rPROP sequences. Acquired signals from a DC sample with both sequences are presented in Fig. 2a, which shows the filtering effect of rPROP as a faster decay in the signal. This decay stems from the free diffusion compartment in the DC sample, and consequently leads to the “tail of the signal” (corresponding to restricted diffusion) appearing for a significantly smaller *q*-value for rPROP ($\approx 37\;{\text{mm}}^{-1}$) than for ST ($\approx 63\;{\text{mm}}^{-1}$).

Furthermore, the following parameters were estimated from both datasets: $D_{0}$, the diffusivity inside a restricted compartment with spherical geometry; ${R_{0}}_{\mathrm{app}}$, the apparent radius of spheres in the sample; and *w*, the fraction of the signal corresponding to a free compartment. Estimates shown in Table 1 were obtained for different ranges of *q*: ${\text{I}}_{1}$—full range; ${\text{I}}_{2}$ and ${\text{I}}_{3}$—*q*-values for which restriction is isolated for rPROP and ST, respectively; and ${\text{I}}_{4}$—low-to-mid *q*-values; and show that rPROP data yielded better estimates than ST for most parameters and *q*-ranges, especially in the low-to-mid *q*-range (i.e. ${\text{I}}_{4}$).

**Table 1:** Estimated parameters for ST and rPROP data from the DC sample employing different ranges of *q*, denoted by ${\text{I}}_{1}$, ${\text{I}}_{2}$, ${\text{I}}_{3}$, and ${\text{I}}_{4}$ (see the text and Figure 2a).

Par.	GT	ST ${\text{I}}_{1}$	rPROP ${\text{I}}_{1}$	ST ${\text{I}}_{3}$	rPROP ${\text{I}}_{2}$	ST ${\text{I}}_{4}$	rPROP ${\text{I}}_{4}$
$D_{0}$	0.63	0.06	0.64	2.8	2.8	2.8	2.8
${R_{0}}_{\mathrm{app}}$	1.90	0.95	1.93	2.2	2.64	4.05	2.86
*w*	0.74	0.74	0.75	0.72	0.74	0.65	0.74

Bold values indicate the waveform (ST or rPROP) whose data yielded estimates closest to the GT based on comparing ST ${\text{I}}_{1}$ with rPROP ${\text{I}}_{1}$, ST ${\text{I}}_{3}$ with rPROP ${\text{I}}_{2}$, and ST ${\text{I}}_{4}$ with rPROP ${\text{I}}_{4}$. Note that for the ${\text{I}}_{1}$ range, the full signal expressions (i.e. also considering the free compartment with $D_{f}=1.4\;\mu {\text{m}}^{2}/\text{ms}$) was employed in the fitting algorithm, while for ranges ${\text{I}}_{2}$, ${\text{I}}_{3}$, and ${\text{I}}_{4}$, data were fit only to the restricted signal expression (Eq. 12). All diffusivities are in $\mu {\text{m}}^{2}/\text{ms}$, and radii in μm. The GT values were obtained through an independent measurement featuring data collected at multiple diffusion times.

Finally, the EAP and MPD for the full range of *q*-values is presented in Fig. 2b. Here, we observe a good representation of the two-compartment system for the distributions obtained from the full rPROP and ST datasets. Additionally, we note that the widths of the narrower lobes in these distributions agrees with the estimated radius obtained from our fitting algorithm.

### Filtering effect in a marginally anisotropic medium

**Figure 3:**
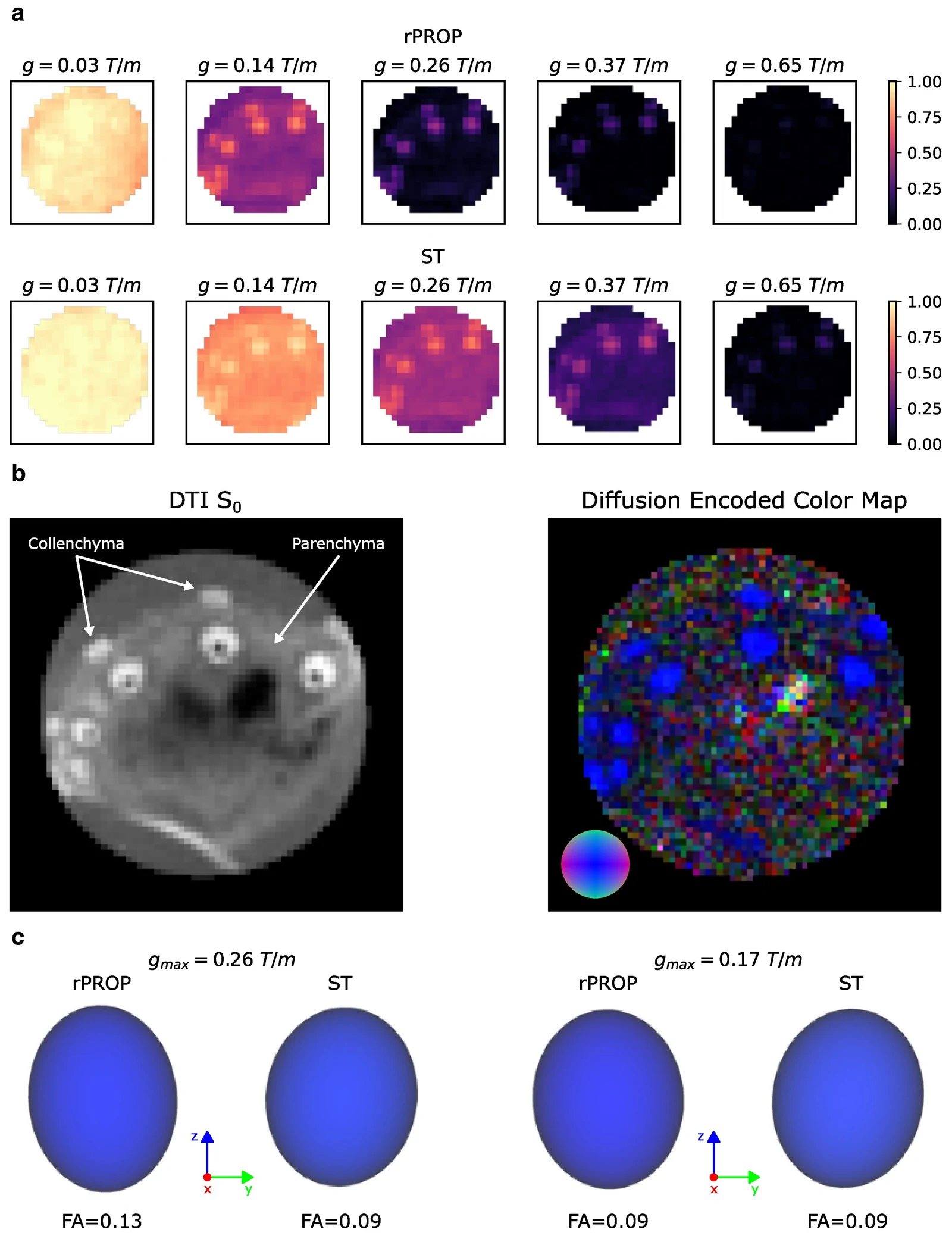
Results of experiments on celery samples. a) Diffusion weighted images obtained via rPROP (top), and ST (bottom) sequences at different gradient strengths. The diffusion encoding gradients were applied in a direction perpendicular to the vascular channels in the celery sample. b) Estimated $S_{0}$ image (left) and DEC map (right) obtained from a traditional DTI protocol (FA values weighting the DEC map are plotted in the range of 0–0.5). c) Different ellipsoids estimated from multidimensional versions of the rPROP and ST sequences, and with two different maximum gradient amplitudes: 0.26T/m ($b_{\mathrm{ST}}\approx 0.38\;\text{ms}/\mu {\text{m}}^{2}$ and $b_{\mathrm{rPROP}}\approx 1.25\;\text{ms}/\mu {\text{m}}^{2}$) and 0.17T/m ($b_{\mathrm{ST}}\approx 0.16\;\text{ms}/\mu {\text{m}}^{2}$ and $b_{\mathrm{rPROP}}\approx 0.52\;\text{ms}/\mu {\text{m}}^{2}$).

To further explore the capability of rPROP to detect restriction, we present analyses on a celery sample. These results are summarized in Fig. 3. First, we show multiple images of a celery sample obtained with rPROP and ST sequences featuring diffusion encoding gradients applied perpendicular to phloem and xylem cells in vascular channels. It can be clearly observed that for the same gradient amplitude, soma regions are attenuated much faster with rPROP than with ST, while still maintaining a fair amount of signal magnitude in the celery’s vascular channels.

To assess the sample’s viability prior to acquiring multidimensional rPROP and ST spectroscopy-like data (i.e. without imaging gradients), a traditional DTI protocol was performed, from which a nondiffusion weighted image ($S_{0}$) and direction encoded color (DEC) map (14) were derived. We show these in Fig. 3b and clearly observe the vascular regions in the celery sample. Furthermore, from the DEC map, we observe that the main diffusion direction in these channels is oriented through the presented slice.

Finally, ellipsoids corresponding to the estimated tensors obtained from the rPROP and ST sequences with two different maximum gradient amplitudes (0.17 and 0.26T/m) are presented in Fig. 3c. These results were obtained with no in-plane spatial encoding, i.e. all parts of the slice contributed to the signal. The main eigenvector direction (i.e. the direction for which diffusion is less restricted) of all four ellipsoids are aligned along the *z*-axis (corresponding to the orientation of the vascular channels); however, the ellipsoids obtained from ST show a slight tilt when compared to the ones from rPROP. The angle between the *z*-axis and main eigenvectors for the ellipsoids with $g_{\max}=0.26\;\text{T}/\text{m}$ was $4.48^{\circ }$ for rPROP, and $7.32^{\circ }$ for ST; and for the ellipsoids with $g_{\max}=0.17\;\text{T}/\text{m}$ it was $3.53^{\circ }$ for rPROP, and $10.93^{\circ }$ for ST. Furthermore, the tensor with the highest fractional anisotropy, FA (which quantifies the degree of diffusion anisotropy), corresponds to the tensor obtained with the rPROP sequence and $g_{\max}=0.26\;\text{T}/\text{m}$ (FA≈0.13), while the other three tensors featured approximately the same value (FA≈0.09).

### Displacement and mean position distributions in mouse SC

**Figure 4:**
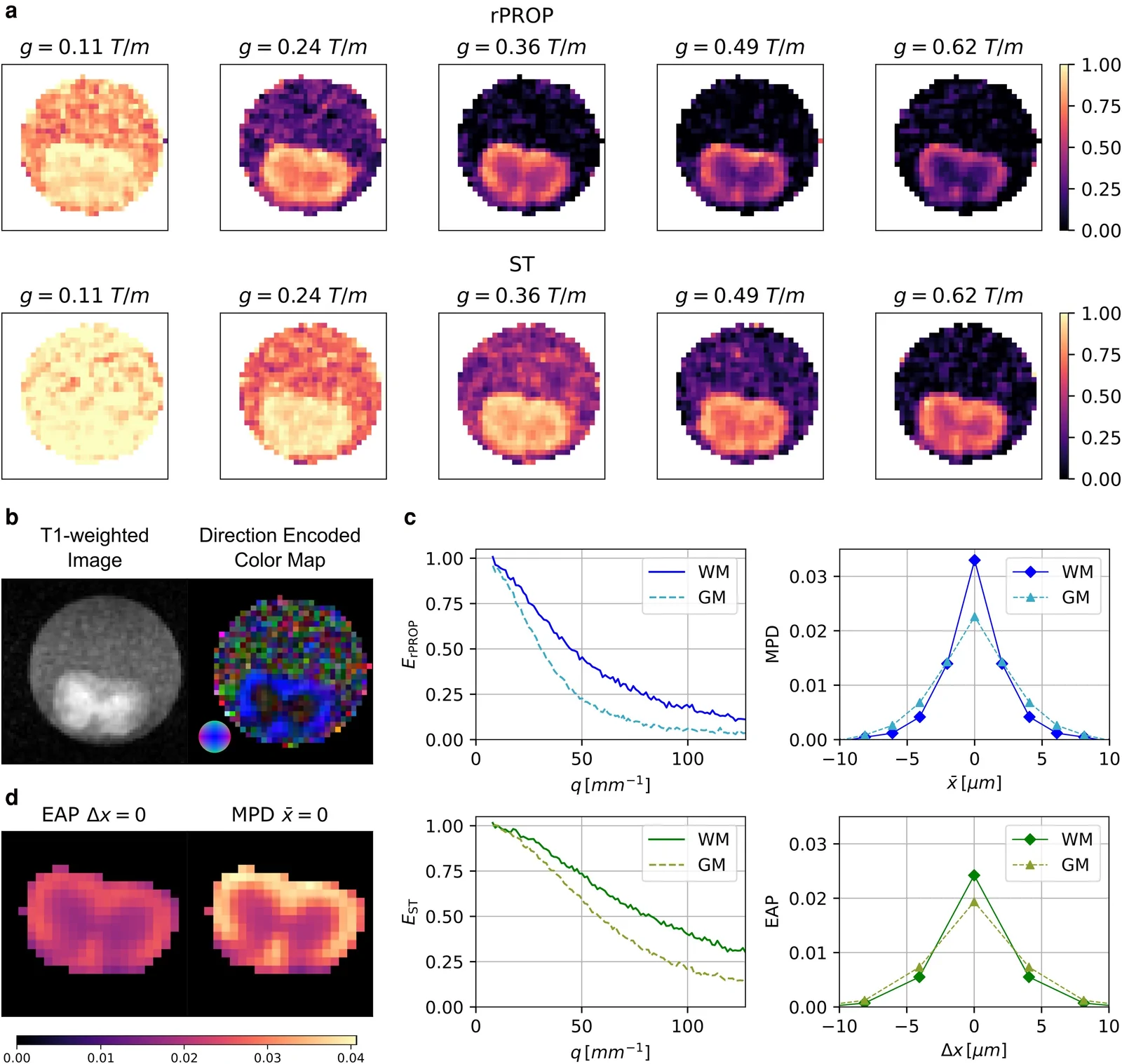
Results of experiments on a SC sample. a) Diffusion weighted images obtained via rPROP (top), and ST (bottom) sequences at different gradient strengths. The diffusion encoding gradients were applied in a direction perpendicular to WM fibers in the SC sample. b) High-res T1-weighted image (left) and DEC map (right) of the sample. The latter was calculated from data obtained via a traditional DTI protocol (FA values weighting the DEC map are plotted in the range of 0–0.6). c) Signal values obtained by averaging data corresponding to WM and GM (left), and the inverse Fourier Transform of the data (right). For rPROP data (top) this operation yields the MPD and for ST data, the EAP. Only the range for which the distributions are non-zero is shown, hence the mismatch of signal and distribution space resolutions. d) EAP (left) and MPD (right) images for Δx=0 and $\bar{x}=0$, respectively. For the EAP, this map is commonly known as the RTOP.

To analyze the SC sample, the EAP and MPD were estimated employing ST and rPROP data, respectively. A series of images of the SC sample obtained from each sequence featuring diffusion encoding gradients applied perpendicular to fibers in white matter are shown in Fig. 4a. Similarly to the DC sample, signal was observed to decay more rapidly for the same gradient amplitude in rPROP images.

A higher-resolution T${\;}_{1}$-weighted image and DEC map (obtained from a traditional DTI protocol) are presented in Fig. 4b, showing regions of the sample corresponding to white matter (WM) and gray matter (GM). Furthermore, from the DEC map, we observe that main diffusion direction in the WM voxels is oriented through the presented slice.

Signal values averaged over WM and GM voxels, and the estimated EAP and MPD are presented in Fig. 4c. From the acquired signals, we observe a faster decay in GM than in WM. The estimated distributions for the latter tissue resemble a single exponentially decaying function with different rates (widths), being broader in the EAP. The signal from rPROP data in GM features a deviation from a mono-exponential function as the *q*-value increases, which could suggest the presence of multiple compartments in this region of the sample. Furthermore, the signal plotted against $q^{2}$ (proportional to the *b*-value) included in the Supplementary material also shows this deviation, as well as the filtering effect in the SC sample. This last feature resembles the results obtained from the DC sample: a faster decay in the signal obtained with rPROP when compared to ST, corresponding to the suppression of free diffusion compartments, followed by a steady decay in the signal, corresponding to compartments featuring restricted diffusion. Finally, the estimated MPD also shows a slight deviation from a Gaussian distribution. Neither of these features is observed in the data nor EAP obtained with the ST sequence.

Finally, maps derived from a voxel-wise analysis are presented in Fig. 4d. Here, we present maps of EAP and MPD values at Δx=0 and $\bar{x}=0$, respectively; for the EAP, this quantity is commonly referred to as the return to origin probability (RTOP) (15). While both maps show contrast between WM and GM, there is a higher difference between tissues for the MPD measure.

## Discussion

### On the pulse sequence

The pulse sequences presented here are special cases of the PROP waveform as shown in Fig. 1a. The PROP sequence is capable of recovering the diffusion propagator in restricted domains (10), which fully describes diffusion processes (16). Furthermore, as shown in Fig. 1b, it contains data from multiple waveforms, such as ST, rPROP, and PSI, which makes any information obtainable from these techniques (e.g. the EAP and MPD) readily accessible from PROP data. While the PROP technique is very powerful, it requires a large amount of samples to take full advantage of this technique. In contrast, the rPROP method presented in this work alleviates this requirement to the same number of samples as other established techniques, such as ST.

In this study, we have shown the filtering capabilities of the rPROP sequence and how they can be exploited to study restricted diffusion. These capabilities can be readily quantified employing the *b*-value (as presented in Theory section) associated with the employed sequence, where we note that $b_{\mathrm{rPROP}}$ (Eq. 11) is larger than $b_{\mathrm{ST}}$ (Eq. 10) for the same gradient amplitude (*q*-value), even for ideal pulse conditions (i.e. δ→0). Furthermore, it is quite remarkable that $b_{\mathrm{rPROP}}$ is not only modulated by the length of the long pulse $\delta_{0}$ but also holds a heavy dependence on the time parameter *τ*. In all the experiments performed in this study, we opted to set this parameter to very short values, however longer ones could be employed to obtain a more effective filter in another realization of the rPROP sequence.

Throughout this study we have compared the rPROP sequence to the ST sequence via different means mainly because of the similarities of the two techniques. As a matter of fact, rPROP is possibly the only waveform which provides the same response as ST to restricted diffusion in common scenarios (see the Supplementary material for more details on the comparison of various gradient waveforms). Moreover, the inverse Fourier transform of the ST and rPROP signals can be similarly interpreted as distributions indicative of the underlying diffusion process in a complementary way. We note that, rPROP and ST also share similarities in that it is possible to perform time-dependent experiments (by varying the diffusion time *Δ*), though such measurements are out of the scope of this study. However, it is worth noting that there are other gradient waveforms which are similar in design to rPROP. For example, the PSI (6), double pulsed field gradient (dPFG) (17–19) waveforms, and a special case of the latter, filter-probe double diffusion encoding (FP-DDE) (20). While these waveforms have been proposed to obtain microstructural information (e.g. anisotropy of the pores), and specifically FP-DDE has been shown to improve SC diffusion imaging compared to traditional methods (21), they do not provide the most efficient free-diffusion filter while maintaining a large amplitude of signal from restricted diffusion. Furthermore, their responses to restricted diffusion deviate from that of ST even in ideal conditions.

**Figure 5:**
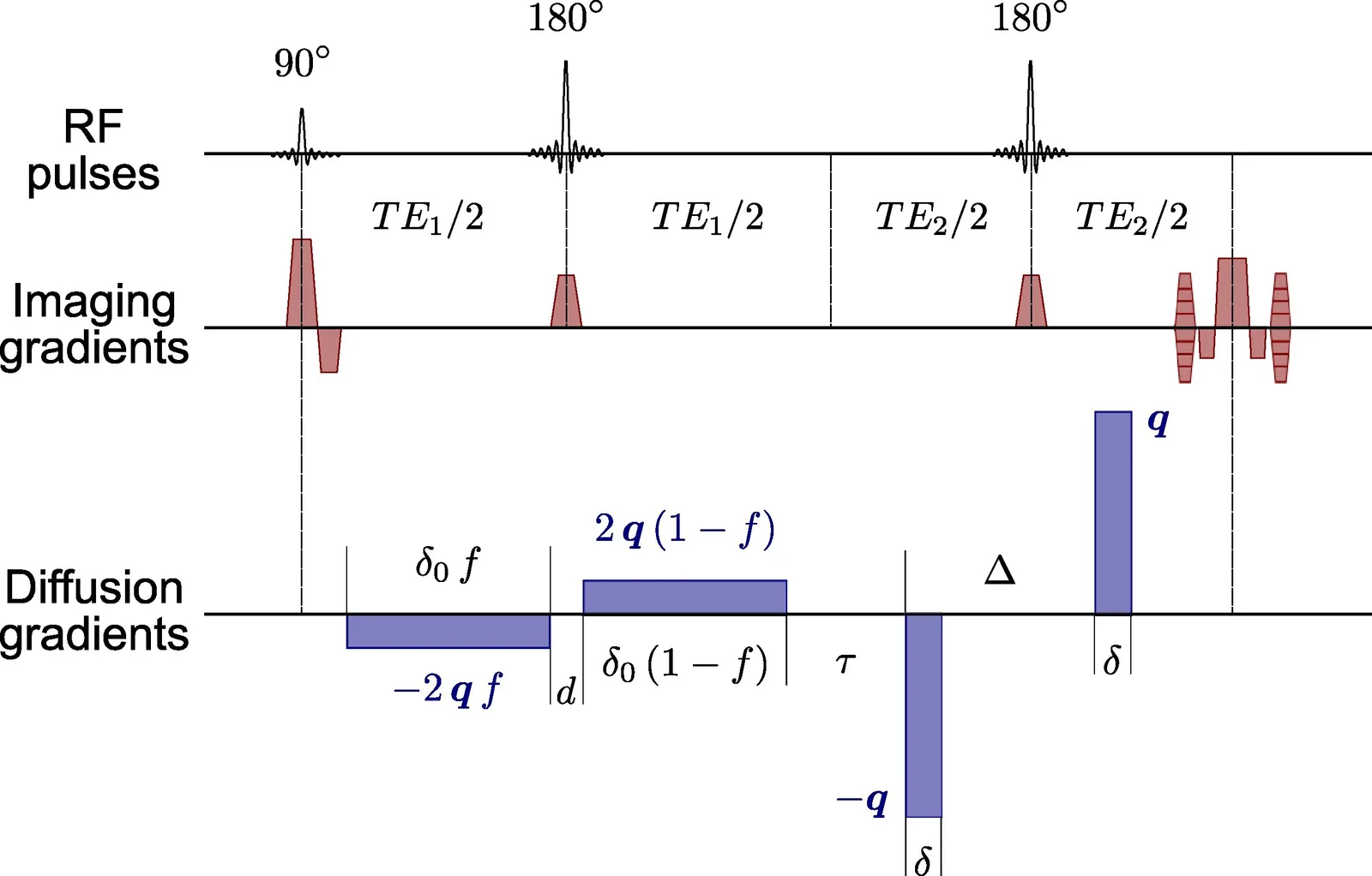
The pulse sequence diagram of the sequence implemented on the benchtop MRI scanner employed to acquire the experimental data. The effective rPROP waveform is obtained by reversing the polarity of the second and third diffusion gradient pulses (due to the 180${\;}^{\circ }$ RF pulses), and is meant to realize the effective rPROP waveform presented in Fig. 1a. The long gradient pulse with total duration $\delta_{0}$ was split into two gradients surrounding a 180${\;}^{\circ }$ RF pulse. The parameter *f* controls the duration of each part of the gradient (separated by a time *d*), *τ* is the time between the long pulse and the first narrow pulse, *δ* is the length of the narrow pulses, and *Δ* is the time between such pulses, traditionally referred to as the diffusion time. The effective echo time, i.e. the time between the excitation pulse and the echo affected by all gradient pulses of this sequence is ${\mathrm{TE}}_{1}+{\mathrm{TE}}_{2}$. In practice, all diffusion gradients were trapezoidal with a ramp-up time of 0.5 ms.

Via different analyses, we have shown advantages of the rPROP sequence over the ST sequence, however it is important to note a couple of the former’s limitations. First, the rPROP sequence will typically require longer echo times (TEs) when compared to the traditional ST sequence because of the additional long pulse. This will result in the signal experiencing prominent T${\;}_{2}$ relaxation. Thus, the rPROP signal features an inherent “T${\;}_{2}$ filter” and a reduction in the signal-to-noise ratio (SNR), which is undesirable if the dynamics of fluids having shorter T${\;}_{2}$ values are to be examined. Secondly, additional RF pulses introduce more complex coherence pathways. For example, in the implemented sequence employed for all the results in this work (see Fig. 5), an additional 180${\;}^{\circ }$ pulse is introduced. In a previous implementation of the sequence, two additional 180${\;}^{\circ }$ pulses were introduced (11). Even though no unwanted artifact (e.g. stimulated echoes) was observed in any of the data acquired in this work, additional care is necessary in the implementations of this sequence. In case this would be an issue in future implementations, phase cycling could be employed as a solution (22); however, it is worth noting that doing so would increase the total acquisition time of the measurement.

The implementation of the sequence used in this work does provide solutions to practical issues. In our implementation, the additional RF pulse of the bipolar gradients reduces the effect of field inhomogeneities. Furthermore, the pulses of the bipolar gradient pair can be asymmetrically applied, i.e. the gradient pulse located prior to the first 180${\;}^{\circ }$ RF pulse can be longer/shorter than the one located after it, so that the shortest TE possible can be achieved. The duration of each gradient pulse is quantified by the parameters $\delta_{0}$, and *f* as introduced in Fig. 5. Finally, the long pulses feature a fairly low gradient magnitude in comparison to the second and third gradient pulses, therefore the effect of concomitant fields (23), which are not perfectly compensated for f≠0.5, is expected to be rather small.

Finally, it is worth noting that, in recent years, enhancing magnetic field gradient performance has been a major focus within the dMR community. For instance, advances in hardware design have enabled clinical scanners to reach gradient amplitudes as high as 0.3 and 0.5T/m (24–26). This ongoing effort to push the limits of gradient hardware is expected to benefit pulse sequences such as ST and rPROP in different ways. For example, larger gradients would allow for narrower pulse widths in ST waveforms, and by consequence a shorter TE, improving measurements where SNR is often a limiting factor. On the other hand, while rPROP features a long initial pulse, this waveform would still benefit from narrower pulses (second and third) to reduce the TE as much as possible. Most importantly, increasing gradient strength would allow for a larger difference between the durations of long and short gradient pulses, especially in clinical scanners, which is crucial for the performance of rPROP. Therefore, rPROP would be well suited to be implemented in these next-generation clinical scanners, in order to take advantage of their high-performance gradient system. Translation to these clinical systems could require modifications in the sequence presented here to first account for field inhomogeneities present in systems with magnetic fields of clinical scanners (3 to 7T) (11). Furthermore, depending on the requirements on *q*- or *b*-values of a study in these systems, it may also be necessary to consider the effect of trapezoidal pulses (27) in the rPROP sequence and framework, namely Eqs. 5, 6, and 11. While the clinical translation of the rPROP technique is one of our main goals, this is left for future works.

### On the fitting results

The results obtained from the DC sample (Table 1) suggest that the parameters estimated from rPROP data approached the ground truth (GT) better when compared to the results obtained via the ST sequence for all but one *q*-range and parameter combination (${R_{0}}_{\mathrm{app}}$ for the ${\text{I}}_{3}$-range). However, there is only a very small difference between estimations for this case in particular. It should be noted that all $D_{0}$ estimates for the reduced *q*-ranges were much higher than the GT value. The GT values of the DC sample were obtained through estimations with an additional rPROP dataset featuring an additional diffusion time (Δ=20ms) as we observed data from this sequence yielded the best fits in a synthetic data analysis. Employing data from a time-dependent measurement offers better sensitivity to compartment’s diffusion coefficients, which would improve the $R_{0}$ estimates as well.

For a different type of analysis, experiments in the long-time regime would result in an apparent diffusion coefficient which scales with the square of the pore radius (22). However, we remark that in our analysis rather than representing a restricted compartment with an apparent diffusivity, we employed the MCF formalism (16, 28, 29) that accounts for all timing parameters of the gradient waveforms we have employed.

### On the anisotropy measurements

The vascular channels in celery can be clearly observed both in the estimated $S_{0}$ image and DEC map in Fig. 3b. Furthermore, these channels feature a high FA value and a main eigenvector (derived from the diffusion tensor) pointing through the presented slice, which confirmed the viability of the celery sample and set an expectation for the tensor to be obtained in spectroscopy-like images. We should note that the DEC map shows a region with high FA around the center of the sample. As shown by the estimated $S_{0}$, this region features a very low signal magnitude (dark), and thus its high FA can be explained by a high noise content.

Detecting anisotropy in spectroscopy-like measurements as done in this work is a very challenging task for any technique, as the anisotropic vascular channels barely constitute ≈2% (volume-wise) or 4% (signal-wise) of the total sample. Thus, it is quite remarkable that the tensor obtained with rPROP featured a higher FA (≈0.13) and maintained the correct main diffusion direction, when compared to the tensor obtained with ST data, even if all computed FA values are fairly low. These values could have been suppressed due to the diffusion time employed in anisotropy measurements (Δ=20ms, yielding a diffusion length of ≈7.5μm), which is not long enough to probe restriction in all parts of the xylem cells (featuring sizes between 16 and 22μm (30)). However, observing an increase of FA with rPROP given this limitation shows the effectiveness of rPROP’s inherent filter in anisotropy measurements.

It is important to note that FA computed from data obtained with smaller gradient amplitudes ($g_{\max}=0.17\;\text{T}/\text{m}$) was very similar for both rPROP and ST sequences (≈0.09), which suggests that there is no apparent systematic error being introduced by the implemented rPROP sequence, which would likely result in an artificial increase of FA.

Finally, it is expected that more comparable results could be obtained had the ST signals been acquired with the same *b*-value rather than the same gradient amplitude as was done here. However, this would require a significant increase in gradient amplitude for ST (approximately twice as large gradients while maintaining the timing parameters following Eqs. 2, 10, and 11), which may not be feasible via standard gradient coils.

### On the estimated distributions

For the DC data, the distributions in Fig. 2b resemble two individual distributions superimposed on each other, one featuring a larger SD (width) than the other. The wider distribution corresponds to the free water compartment, while the narrower one to the restricted compartment (sphere). Furthermore, the width of the latter and the estimated radius obtained via model fitting agree very well with one another for both distributions when employing full datasets (${\text{I}}_{1}$). We note that for the EAP, the width of the narrow lobe corresponds to the diameter of the sphere, which is the maximum displacement water molecules can traverse. This observation has been previously exploited to successfully study compartment sizes in yeast cells (4). On the other hand, in the MPD, the width of the narrow lobe corresponds to the radius of the sphere. This is because, in a reference frame whose origin coincides with the center of the sphere, the mean position molecules can occupy is bounded by the radius.

Both the EAP and MPD are useful to study samples which do not feature well-defined compartments, as no assumption on the shape of the compartments is made when performing the analysis. For the SC data, the distributions obtained with both rPROP and ST from WM feature a steady decay as the mean position and displacement (respectively) increase. We note that the ST signal for this tissue has not fully decayed at the highest *q*-value acquired in our experiments, which can introduce widening effects in the estimated displacement distribution. This effect is less severe for rPROP data, where the signal has been almost nulled by the end of the measurement as a consequence of the larger diffusion weighting. In both displacement and mean position distributions, GM features a larger SD when compared to WM. However, the differences between both tissues are more apparent for the MPDs obtained through rPROP. Considering there are multiple compartments in WM and GM, it was expected to observe distributions (EAP and MPD) deviating from Gaussian ones. However, we only observed slight deviations in our results, which is likely due to additional processes taking place in tissue, such as water exchange. This process would yield wider distributions and harder-to-distinguish compartments (17, 31).

It is important to note that the estimated distributions from both ST and rPROP data are limited by the resolution in displacement and mean position spaces, directly defined by the maximum *q*-value employed to acquire the data. Techniques such as zero-padding or estimating the distributions at additional Δx and $\bar{x}$ points in the Fourier Transform could be explored to obtain finer, yet interpolated, distributions. However, we decided against this to avoid introducing any artifactual trends in the distributions that could lead to their misinterpretation.

### Extensions

The presented rPROP measurements can be readily extended in order to access additional information. For example, data can be collected by sampling the 3D *q*-space, and transformed in a way analogous to what is done via ST measurements (15, 32). This way, the rPROP measurements could yield a new 3D MPD, which can provide further insight into a specimen’s microstructure. For example, the mean apparent propagator MRI (MAP-MRI) method (15) features a host of scalar measures derived from the heavily attenuated portions of the three-dimensional ST signal decay profile and the estimated EAP. A similar analysis can be performed with the rPROP signal, whose inherently strong ability to suppress free diffusion as well as the different meaning of the MPD to be reconstructed could boost the sensitivity of such scalar measures to the underlying microstructural alterations.

We highlight that with rPROP data it is possible to obtain relevant quantities (such as the MPD) without relying on assumptions on the underlying microstructure of the samples to investigate. Therefore, reconstruction of the MPD via a Fourier Transform can be considered a model-free approach, similar to the reconstruction of EAP via diffusion spectrum imaging (DSI) (32). However, and while performing analyses employing previously proposed models with rPROP data is out of the scope of this study, here we discuss certain benefits this gradient waveform may bring in this context. Owing to rPROP’s pronounced sensitivity to restricted diffusion and its effective attenuation of free-diffusion contributions, incorporating models such as NODDI (33), the standard model (34), and SANDI (35) could enable improved estimation of restriction-dependent microstructural parameters while mitigating confounding effects from free-diffusion components. NEXI (36) is another such method that also accounts for water exchange. Indeed, water exchange has been studied via ST and dPFG sequences. Using the latter, it is of interest to incorporate the exchange between almost-restricted and free diffusion domains (37). Another approach has envisioned the first pulse pair as a filter to enhance contrast between compartments with different diffusivities for probing exchange between them (38). Because rPROP excels in creating contrast between free and restricted domains, a further improvement may be possible, e.g. by varying diffusion time and/or other timing parameters, and developing associated models. However, this is outside the scope of this study.

Here, we employed a multidimensional protocol with the goal of detecting anisotropy (Detecting anisotropy in predominantly isotropic samples section). We note that new tensors can be accessed from such measurements. We can, for example, define mean squared displacement and mean position tensors from multidimensional measurements employing the ST and rPROP sequences, respectively. Combining the unique components from these two tensors allows us to estimate a new tensor whose components are the autocorrelation of particles’ initial positions, and another whose components denote the correlation between initial and end positions during a measurement. With some additional manipulation, the autocorrelation parameters can be combined to compute the moment of inertia tensor, which provides information regarding the resistance of a certain compartment to be rotated about an arbitrary axis. The details of this scheme are provided in the Supplementary material. Furthermore, as is the case with traditional DTI measurements, invariants such as fractional anisotropy and trace can be easily computed from these tensors. Given that most MRI protocols already feature a DTI-like measurement, we highlight that in order to access these additional quantities, only one dataset should be added to said protocol: a multidimensional measurement with the rPROP sequence, whose acquisition time would be comparable to that of traditional DTI.

Finally, acknowledging the requirement of longer TEs with rPROP, we also note that the implementation of rPROP in (pre-)clinical scanners is not expected to be a complicated task to perform. As we have shown throughout this work, gradient amplitudes required to perform different analyses with rPROP are significantly lower than those required with ST, resulting in a reduction in hardware requirements and a more accessible sequence to use in scanners with lower specifications.

### Limitations on the study

This works has two main limitations that warrant discussion. First, the potential benefits of the rPROP method were assessed through the conceptual analyses and experimental findings as presented in the main text and Supplementary material. Other methods such as microscopy, phantom measurements, and numerical simulations (39, 40) could be used for validating the rPROP method and investigating quantitative correlations between the rPROP-derived metrics and quantities obtained via such independent methods. However, each of these techniques introduces its own challenges and potential sources of uncertainty. For example, histological validation requires tissue fixation, sectioning, dehydration, and mounting, all of which can substantially alter the native hydrated microstructure probed by diffusion MRI. Employing such extensive validation methods is left for future studies as they are beyond the intended scope of this technical manuscript, which is focused on the conceptual benefits of the method.

Secondly, the reproducibility our results could be improved by acquiring data from additional samples than the ones shown here. Such experiments would make it possible to perform additional statistical analyses to the results obtained with rPROP, and further showcase its difference with respect to traditional ST measurements. We remark, however, that in the Supplementary material we have included results for two additional SC samples, whose data yielded the same features observed in the sample presented in this main article, supporting rPROP’s reproducibility in independent samples.

## Conclusion

In conclusion, we introduced a new diffusion encoding pulse sequence, here referred to as rPROP, and showed its advantages over traditional ST measurements at probing restricted diffusion, which holds various properties that characterize complex specimens. This occurs thanks to rPROP’s high diffusion weighting, which is employed as a free diffusion filter for multi-compartment systems. We showed this filtering effect by estimating restriction-related parameters (size of restricted compartments) in data at different ranges of *q*-values (gradient amplitudes), where fits obtained with rPROP data outperformed the ones from ST data in most cases. Furthermore, we introduced a method to further exploit rPROP’s filtering capabilities by employing a scheme akin to diffusion tensor imaging to detect traces of anisotropy in environments featuring mostly isotropic content. The tensor obtained with rPROP showed an increased value of fractional anisotropy when compared to the tensor obtained with a traditional DTI measurement in a sample composed of ≈98% isotropic content. Finally, we presented a distribution accessible from rPROP data, the MPD, and showed how it differs from the EAP (attainable from ST acquisitions) in a water-emulsion system for different *q*-value ranges, and in a SC sample. Therefore, we propose the rPROP measurement as a viable alternative to ST measurements in characterization studies.

## Theory

### The diffusion propagator and related distributions

The diffusion propagator can be interpreted as the probability of a particle (or a bundle of them) to move from an initial position (r) to a final one ($r^{\prime }$) within a certain time *Δ*, and is written as $p(r^{\prime },\Delta |r)$. In a recent publication (10), a diffusion encoding pulse sequence capable of extracting the diffusion propagator from MR measurements was presented. The gradient waveform of this sequence is shown in Fig. 1a, and its signal expression can be written as


(1) $$E\left(q,q^{\prime }\right)=\int_{\Omega }\;\text{d}r\;\rho (r)\int_{\Omega }\;\text{d}r^{\prime }\;p\left(r^{\prime },\Delta |r\right)\;e^{-i2\pi \left(q\cdot r+q^{\prime }\cdot r^{\prime }\right)},$$

where r and $r^{\prime }$ are the vectors denoting the positions of the particles, respectively, during the first and the second narrower pulses in a frame of reference whose origin is established by the average position of the particle during the application of the long pulse. The above expression is valid for restricted domains as well as for free diffusion in the case when the second and third pulses are infinitesimally short and the first pulse is long enough so that the average position encoded by the first pulse is the position of the pore’s center of mass (10). In Eq. 1, q is defined as


(2) $$q(t)=\frac{\gamma }{2\pi }\int_{0}^{t}\;\text{d}t^{\prime }g(t^{\prime }),$$

where, *γ* is the gyromagnetic ratio and g(t) is the time-dependent magnetic field gradient vector. We note that by employing this definition, q has the units of inverse length.

As shown in Ref. (10), the acquired data from this sequence for different $\left(q,q^{\prime }\right)$ samples can be transformed to obtain the diffusion propagator for closed pores. However, sampling only certain trajectories on the $qq^{\prime }$ plane can lead to some well-known sequences, and yield different quantities, as illustrated in Fig. 1b. For example, acquiring data for $q^{\prime }=-q$ yields the traditional ST sequence (1), which can extract the EAP ($\hat{p}$), i.e. the probability distribution of net displacements. Acquiring data for $q^{\prime }=q$ yields a new distribution, which we present in this work and refer to as the MPD ($\bar{\;p}$).

Here, we highlight the Fourier relation between these distributions and their corresponding diffusion encoded signals. For narrow second and third gradient pulses in Fig. 1a, and for $q^{\prime }=-q$, the signal expression obtained for different q-vectors is


(3) $$E_{\mathrm{ST}}(q)=\int_{\Omega }\;\text{d}R\;e^{-i2\pi q\cdot R}\;\hat{p}(R),$$

where $R=r^{\prime }-r$ is the net displacement vector, and $\hat{p}(R)$ is the EAP or displacement distribution (1, 4), i.e. the probability of particles to have a net displacement R over time *Δ* given by


(4) $$\hat{p}(R)=\int_{\Omega }\;\text{d}r\;\rho (r)\;p\left(r+R,\Delta |r\right).$$

This distribution is accessible via $F^{-1}[E_{\mathrm{ST}}(q)]$ (where $F^{-1}$ denotes the inverse Fourier Transform). Similar to k-space in MR imaging, the reconstructed propagator has been viewed as a “q-space image” representing the displacements (41).

Similarly, employing the narrow pulse assumption, but for $q^{\prime }=q$, i.e. the rPROP sequence, the signal expression is


(5) $$E_{\mathrm{rPROP}}(Q)=\int_{\Omega }\;\text{d}\bar{r}\;e^{-i2\pi Q\cdot \bar{r}}\;\bar{p}(\bar{r})$$

with


(6) $$\bar{p}(\;\bar{\;r})=2^{d}\int_{\Omega }\;\text{d}r\;\rho (r)\;p\left(2\;\bar{\;r}-r,\Delta |r\right),$$

where we defined Q=2q and *d* is the number of spatial dimensions in the measurement. Here, $\bar{r}=(r+r^{\prime })/2$ is the mean position vector, and $\bar{p}(\bar{r})$ is the MPD, i.e. the probability of particles to acquire a mean position $\bar{r}$, which is defined to be the average of the two positions at the beginning and end of the diffusion time *Δ*. A Fourier relation between $E_{\mathrm{rPROP}}(Q)$ and $\bar{p}(\bar{r})$ can also be observed in Eq. 5, thus this probability distribution, or the “Q-space image” is directly accessible via $F^{-1}[E(Q)]$.

### The two-compartment model

Data from a system composed of two nonexchanging compartments were simulated to assess the filtering capability of the rPROP waveform. The two compartments comprising this system were a free diffusion pool of water and a spherical compartment with radius $R_{0}$. Given that exchange is not being considered in this simulation, the total signal obtained from this system can be simply calculated via


(7) $$E_{\mathrm{sys}}=w\;E_{\mathrm{free}}+(1-w)\;E_{\mathrm{rest}},$$

where *w* is the fraction of free water in the system, and $E_{\mathrm{free}}$ and $E_{\mathrm{rest}}$ are the signals obtained from a free diffusion compartment and a fully restricted one, respectively. The signal from the first compartment was calculated via


(8) $$E_{\mathrm{free}}=\exp\;\left(-b\;D_{f}\right),$$

where $D_{f}$ is the diffusion coefficient of the freely diffusing water, and *b* is the *b*-value, commonly employed to describe diffusion weighting. *b* can be generally defined for any gradient waveform g(t) through the expression (42)


(9) $$b=\gamma^{2}\int_{0}^{TE}\;\text{d}t\;q^{2}(t),$$

where TE is time of echo of any given pulse sequence. For the ST sequence, Eq. 9 yields a value of


(10) $$b_{\mathrm{ST}}=4\pi^{2}q^{2}\;(\Delta -\delta /3),$$

where *δ* is the gradient pulse duration, and *Δ* is the diffusion time. The *b*-value for the rPROP sequence can be derived and numerically computed employing a method presented in Ref. (43). For the sequence realization illustrated in Fig. 5, the *b*-value is


(11) $$b_{\mathrm{rPROP}}=\frac{4}{3}\pi^{2}q^{2}\left(12\;d\;f^{2}+5\delta +3\Delta +4\delta_{0}+12\tau \right),$$

where *d* is the separation between the pulses after splitting the long pulse having duration $\delta_{0}$, into a pair of bipolar gradients; the fraction *f* denotes the fractional duration of the first part of the long gradient; *τ* is the time between the end of the long pulse and start of the first shorter pulse; *δ* is the duration of the narrower pulses; and *Δ* is the diffusion time.

The signal from spheres ($E_{\mathrm{rest}}$) was calculated employing a multiple correlation function (MCF) formalism (16, 28). The unknown parameters involved in the signal expressions are *w*, $D_{0}$ (dominant in the free diffusion regime), and $R_{0}$ (dominant in the restricted diffusion regime).

**Figure 6:**
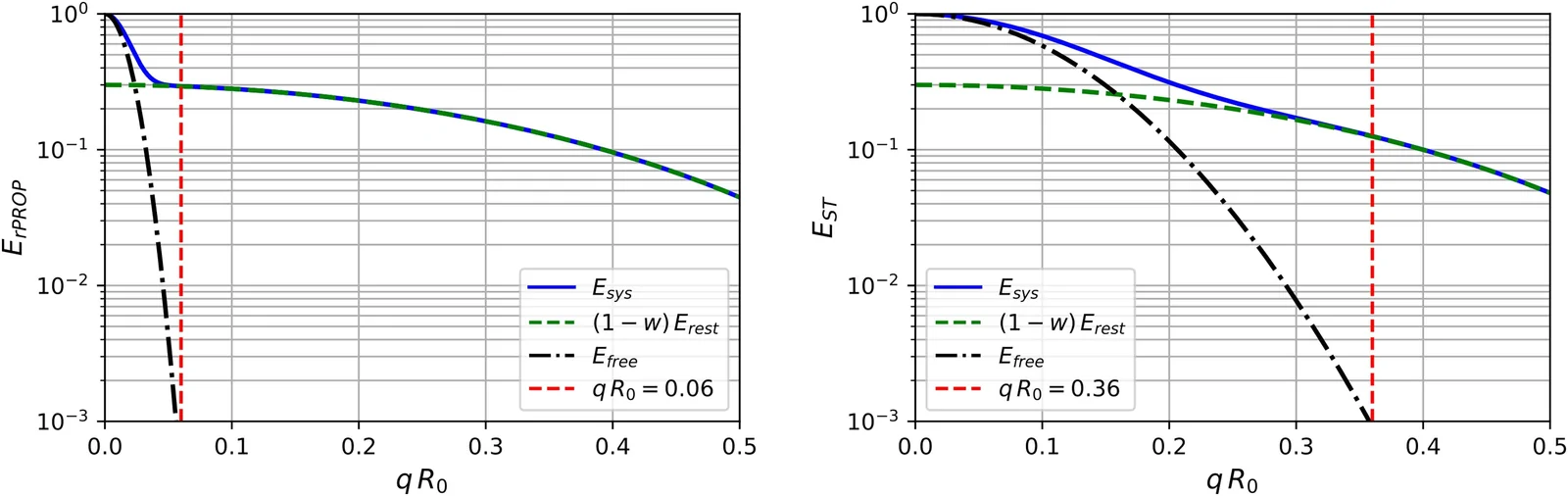
Example of a signal profile obtained from a two-compartment system featuring free diffusion and restricted diffusion within a sphere. To the left is the rPROP signal, and to the right is the ST signal, both of which were obtained with the same range of *q*-values, here plotted as the dimensionless variable $q\;R_{0}$. The solid curve is the simulated signal, $E_{\mathrm{sys}}$, obtained from Eq. 7; the dashed curve is the restricted diffusion part of the signal, i.e. $(1-w)\;E_{\mathrm{rest}}$, obtained with an MCF approach (29); the dashed-dotted curve is the signal from the free compartment, $E_{\mathrm{free}}$, obtained from Eq. 8; and the vertical dashed line delimits the region of the signal where $E_{\mathrm{sys}}\approx (1-w)\;E_{\mathrm{rest}}$. Note that the fully restricted signal can be isolated (i.e. $E_{\mathrm{free}}\approx 0.001$) from $q\;R_{0}\approx 0.06$ employing rPROP, and from $q\;R_{0}\approx 0.36$ with ST. The simulation parameters were: $D_{0}=D_{f}=2\;\mu {\text{m}}^{2}/\text{ms}$, $R_{0}=5\;\mu \text{m}$, and w=0.7. The acquisition parameters were: Δ=17.76ms, δ=2ms for both ST and rPROP, and $\delta_{0}=500\;\text{ms}$, and τ=2ms for rPROP only.

Two different *q*-ranges can be identified in the simulated signals: for low *q*-values, the free component will dominate, whilst for high ones the restricted signal will dominate. Therefore, for appropriate values of *q*, it is possible to properly represent the system’s signal just by its restricted component, leading to the following approximation:


(12) $$E_{\mathrm{sys}}\approx (1-w)\;E_{\mathrm{rest}},$$

which can be used to directly estimate the unknown parameters of the system with only a subset of data, emphasizing the restriction parameter, $R_{0}$. An example of these ranges is illustrated in Fig. 6, which was generated considering $D_{0}=D_{f}=2\;\mu {\text{m}}^{2}/\text{ms}$, $R_{0}=5\;\mu \text{m}$, and w=0.7.

In this example, we can clearly observe that the rPROP sequence suppresses the free diffusion contribution to the signal at much lower *q*-values when compared to the traditional ST sequence. This can be quantified by the transition or inflection point, which can be noted in Fig. 6, located at $q\;R_{0}=0.06$ for rPROP, and at $q\;R_{0}=0.36$ for ST. This shows a 6-fold reduction in gradient requirement to suppress the free diffusion compartment when using rPROP. It should be noted that this inflection point has been previously suggested as a powerful parameter to study in microstructure analyses (44) and such frameworks could benefit from rPROP since it makes the inflection point significantly more conspicuous which can also be observed in Fig. 6. A third range of *q* can then be identified, corresponding to *q*-values where the free diffusion compartment has been nullified with rPROP sequence, but not with the ST one. This range is meant to represent the reduction in gradient amplitude required to characterize restricted diffusion achievable with the rPROP sequence.

## Methods

### Estimation of the system’s parameters in experimental data

To assess whether restriction parameters can be estimated from partial ST and rPROP signals, data was acquired employing the pulse sequence illustrated in Fig. 5 and the traditional ST waveform, both of which were implemented on a 0.55T benchtop MRI system (*PureDevices*, Rimpar, Germany). A DC sample (*Arla Foods*, Solna, Sweden) was scanned with this type of analysis. This sample features fat globules (micro-spheres), with an average diameter of ≈4μm (45), which allows for employing a model such as the one presented in Eq. 7 in the estimation algorithm.

For both ST and rPROP data, 100 different *q*-values were acquired with a $q_{\min}$ of $7.96\;{\text{mm}}^{-1}$ and a $q_{\max}$ of $127.3\;{\text{mm}}^{-1}$, with an equal spacing between samples. The corresponding minimum and maximum gradient amplitudes were 0.09 and 1.49T/m, respectively. Gradient pulse durations and diffusion times were the same for both sequences, with δ=2ms and Δ=30ms, while the additional timing parameters for rPROP were $\delta_{0}=35\;\text{ms}$, τ=1ms, and f=0.5. All gradients featured a trapezoidal shape with a ramp-up time of 0.5 ms. The employed echo times (TE), repetition times (TR) and number of averages were: 75ms, 2s, and 100 for both sequences. We note that the GT for this analysis was defined using additional data with a shorter diffusion time (Δ=20ms) along with the original data to estimate $D_{0}$, $R_{0}$, and *w*. The estimated parameters used as GT were: $D_{0}=0.639\;\mu {\text{m}}^{2}/\text{ms}$, $R_{0}=1.9\;\mu \text{m}$, and w=0.74. Note that the diffusion coefficient of the free compartment was set to $D_{f}=1.4\;\mu {\text{m}}^{2}/\text{ms}$, which provided the smallest residuals after performing fits to data with multiple diffusion times, for all estimations.

The acquired ST and rPROP data were fit to Eq. 7 employing different ranges of *q*. These ranges were: ${\text{I}}_{1}$—full range; ${\text{I}}_{2}$ and ${\text{I}}_{3}$—high *q*-values for ST ($q\geq 63\;{\text{mm}}^{-1}$) and rPROP ($q\geq 37\;{\text{mm}}^{-1}$), which correspond to *b*-values yielding $E_{\mathrm{free}}=\exp\;(-b_{\mathrm{ST}/\text{rPROP}}\;D_{f})\leq 0.002$; and ${\text{I}}_{4}$—low-to-mid *q*-values ($q\in [37,63]\;{\text{mm}}^{-1}$). The fits obtained from range ${\text{I}}_{4}$ were compared to the ones from ${\text{I}}_{1}$, ${\text{I}}_{2}$, and ${\text{I}}_{3}$, as well as the estimated GT, to assess whether low-to-mid *q*-values are sufficient to accurately extract restriction information from either ST and rPROP data. Fits were obtained with the least_squares function of the scipy package, restricting the unknown parameters to positive values and imposing upper limits to *w* and $D_{0}$ to 1 and $3.1\;\mu {\text{m}}^{2}/\text{ms}$, respectively.

### Detecting anisotropy in predominantly isotropic samples

The filtering capabilities of the rPROP sequence can also be employed for the detection of anisotropic environments at lower gradient amplitudes. This is especially beneficial in samples featuring a high contribution of isotropic compartments, which are largely suppressed in rPROP measurements. To test this hypothesis, a celery sample was investigated. Such samples have been previously employed to study anisotropic diffusion as it features well-defined areas in which diffusion is either isotropic (soma) or anisotropic (xylem) (30, 46).

To mimic a system where diffusion is predominantly free, but we know has anisotropic compartments, spectroscopy-like data (i.e. without frequency nor phase encoding gradients) was analyzed. Spectroscopy-like measurements can be interpreted as the averaged signal over a certain region of the celery sample, mimicking a system composed mostly of regions with isotropic diffusion. In total, four spectroscopy-like datasets were acquired: the first two featured a maximum gradient amplitude ($g_{\max}$) of 0.26T/m for both ST and rPROP; and the remaining two, a maximum gradient amplitude of 0.17T/m for both pulse sequences. All datasets featured the same minimum gradient amplitude ($g_{\min}$) of 0.053T/m and the same direction distribution: 6 directions for the low gradient amplitude, and 21 directions for larger gradient amplitudes. The diffusion encoding directions were arranged based on tessellation of an icosahedron on the hemisphere (see Supplementary material for the full set of directions).

Gradient pulse lengths and diffusion times were the same for both sequences, with δ=2ms and Δ=20ms, while the additional timing parameters for rPROP were $\delta_{0}=30\;\text{ms}$, τ=3ms, and f=0.5. All gradients featured a trapezoidal shape with a ramp-up time of 0.5 ms. The employed echo times (TE), repetition times (TR), and number of averages were: 55ms, 1s, and 20 for both sequences.

The diffusion tensor of each dataset was then estimated, from which their fractional anisotropy (FA) was finally calculated. The angle between the main eigenvectors (corresponding to the largest eigenvalue) of these tensors and the *z*-axis (which corresponds, on average, to the direction of the xylem) was additionally calculated in order to qualify the alignment of each tensor. This angle was calculated following: $\text{arccos}({\hat{e}}_{1}\cdot \hat{k})$, where ${\hat{e}}_{1}$ is the tensor’s main eigenvector and $\hat{k}$ is the unit vector along the *z*-axis.

### Estimation of the displacement and mean position distributions

The mean displacement and mean position distributions were estimated from the DC data and the full *q*-range (${\text{I}}_{1}$ in Estimation of the system’s parameters in experimental data section). An in-house implementation of the inverse Fourier Transform was employed to compute the EAP and MPD. These distributions were additionally studied in data from an excised mouse SC sample obtained as part of an unrelated Huntington’s disease study following national and local ethical guidelines (ethical permit number 14741-2019). For this, data employing both ST and rPROP sequences were acquired. All images featured a matrix size of 35×35 and an in-plane voxel size of $\approx 0.14\times 0.14\;{\text{mm}}^{2}$. Furthermore, all diffusion gradients were applied along a direction perpendicular to the fibers in white matter tissue. The *q*-values applied in this study ranged from $0.09\;{\text{mm}}^{-1}$ to $127.32\;{\text{mm}}^{-1}$ (corresponding to a maximum gradient amplitude of ≈1.49T/m) with 100 evenly spaced samples. Gradient pulse lengths and diffusion times were set the same for both sequences, with δ=2ms and Δ=10ms, while the additional timing parameters for rPROP were $\delta_{0}=10\;\text{ms}$, τ=1ms, and f=0.5. All gradients featured a trapezoidal shape with a ramp-up time of 0.5 ms. The employed echo time (TE), repetition time (TR), and number of averages were: 27.2ms, 400ms, and 40 for both ST and rPROP. Data acquired from the scanner was then used to estimate the MPD and the EAP via an in-house implementation of the inverse Fourier Transform. All computations were performed using MATLAB (*Mathworks Inc.*, Natick, USA) and custom Python (version 3.12.3) scripts.

## Supplementary Material

pgag293_Supplementary_Data

## Data Availability

The data and analysis scripts used throughout this article and its related Supplementary material are available in the following repository: https://github.com/alfor07/rPROP.

## References

[1] Stejskal EO, Tanner JE. 1965. Spin diffusion measurements: spin echoes in the presence of a time-dependent field gradient. J Chem Phys. 42:288–292.

[2] Basser P, Mattiello J, LeBihan D. 1994. MR diffusion tensor spectroscopy and imaging. Biophys J. 66:259–267. 10.1016/S0006-3495(94)80775-1 PMC1275686 8130344

[3] Grebenkov DS . 2008. Laplacian eigenfunctions in NMR. I. A numerical tool. Concepts Magn Reson A. 32A:277–301.

[4] Cory DG, Garroway AN. 1990. Measurement of translational displacement probabilities by NMR: an indicator of compartmentation. Magn Reson Med. 14:435–444. 10.1002/mrm.1910140303 2355827

[5] Telkki V-V, Jokisaari J. 2009. Determination of the structure of wood from the self-diffusion probability densities of a fluid observed by position-exchange NMR spectroscopy. Phys Chem Chem Phys. 11:1167–1172. 10.1039/b817727a 19209359

[6] Laun FB, Kuder TA, Semmler W, Stieltjes B. 2011. Determination of the defining boundary in nuclear magnetic resonance diffusion experiments. Phys Rev Lett. 107:048102. 10.1103/PhysRevLett.107.048102 21867047

[7] Kärger J, Heink W. 1983. The propagator representation of molecular transport in microporous crystallites. J Magn Reson. 51:1–7.

[8] Laun FB, Kuder TA, Wetscherek A, Stieltjes B, Semmler W. 2012. NMR-based diffusion pore imaging. Phys Rev E. 86:021906. 10.1103/PhysRevE.86.021906 23005784

[9] Özarslan E . 2021. Recovering almost everything diffusion could reveal. arXiv:2105.00145.

[10] Özarslan E, et al 2023. Diffusion within pores fully revealed by magnetic resonance. J Chem Phys. 158:161102. 10.1063/5.0146304 37093135

[11] Ordinola Santisteban AM, Özarslan E. 2021. Magnetic resonance measurement and reconstruction of the diffusion propagator. arXiv:2106.16181.

[12] Mattiello J, Basser PJ, Le Bihan D. 1997. The b matrix in diffusion tensor echo-planar imaging. Magn Reson Med. 37:292–300. 10.1002/mrm.1910370226 9001155

[13] Beaulieu C . 2002. The basis of anisotropic water diffusion in the nervous system – a technical review. NMR Biomed. 15:435–455. 10.1002/nbm.782 12489094

[14] Pajevic S, Pierpaoli C. 1999. Color schemes to represent the orientation of anisotropic tissues from diffusion tensor data: application to white matter fiber tract mapping in the human brain. Magn Reson Med. 42:526–540. 10467297

[15] Özarslan E, et al 2013. Mean apparent propagator (MAP) MRI: a novel diffusion imaging method for mapping tissue microstructure. Neuroimage. 78:16–32. 10.1016/j.neuroimage.2013.04.016 PMC4059870 23587694

[16] Grebenkov DS . 2007. NMR survey of reflected Brownian motion. Rev Mod Phys. 79:1077–1137.

[17] Cory DG, Garroway AN, Miller JB. 1990. Applications of spin transport as a probe of local geometry. Polym Prepr. 31:149.

[18] Callaghan PT, Manz B. 1994. Velocity exchange spectroscopy. J Magn Reson Ser A. 106:260–265.

[19] Callaghan PT, Komlosh ME. 2002. Locally anisotropic motion in a macroscopically isotropic system: displacement correlations measured using double pulsed gradient spin-echo NMR. Magn Reson Chem. 40:S15–S19.

[20] Skinner NP, Kurpad SN, Schmit BD, Budde MD. 2015. Detection of acute nervous system injury with advanced diffusion-weighted MRI: a simulation and sensitivity analysis. NMR Biomed. 28:1489–1506. 10.1002/nbm.3405 26411743

[21] Skinner NP, et al 2018. Filter-probe diffusion imaging improves spinal cord injury outcome prediction. Ann Neurol. 84:37–50. 10.1002/ana.25260 PMC6119508 29752739

[22] Callaghan PT . Principles of nuclear magnetic resonance microscopy. Oxford University Press, 1991.

[23] Bernstein MA, et al 1998. Concomitant gradient terms in phase contrast MR: analysis and correction. Magn Reson Med. 39:300–308. 10.1002/mrm.1910390218 9469714

[24] Setsompop K, et al 2013. Pushing the limits of *in vivo* diffusion MRI for the human connectome project. Neuroimage. 80:220–233. 10.1016/j.neuroimage.2013.05.078 PMC3725309 23707579

[25] Foo TKF, et al 2020. Highly efficient head-only magnetic field insert gradient coil for achieving simultaneous high gradient amplitude and slew rate at 3.0T (MAGNUS) for brain microstructure imaging. Magn Reson Med. 83:2356–2369. 10.1002/mrm.28087 31763726

[26] Huang SY, et al 2021. Connectome 2.0: developing the next-generation ultra-high gradient strength human MRI scanner for bridging studies of the micro-, meso- and macro-connectome. Neuroimage. 243:118530. 10.1016/j.neuroimage.2021.118530 PMC8863543 34464739

[27] Mattiello J, Basser PJ, LeBihan D. 1994. Analytical expressions for the b-matrix in NMR diffusion imaging and spectroscopy. J Magn Reson A. 108:131–141.

[28] Barzykin AV . 1999. Theory of spin echo in restricted geometries under a step-wise gradient pulse sequence. J Magn Reson. 139:342–353. 10.1006/jmre.1999.1778 10423371

[29] Özarslan E, Shemesh N, Basser PJ. 2009. A general framework to quantify the effect of restricted diffusion on the NMR signal with applications to double pulsed field gradient NMR experiments. J Chem Phys. 130:104702. 10.1063/1.3082078 PMC2736571 19292544

[30] Özarslan E, Komlosh ME, Lizak MJ, Horkay F, Basser PJ. 2011. Double pulsed field gradient (double-PFG) MR imaging (MRI) as a means to measure the size of plant cells: double-PFG MRI to measure the size of plant cells. Magn Reson Chem. 49:S79–S84. 10.1002/mrc.2797 PMC3608120 22290713

[31] Williamson NH, Ravin R, Cai TX, Rey JA, Basser PJ. Hydrophysiology NMR reveals mechanisms of steady-state water exchange in neural tissue. bioRxiv 2024.12.12.628254, preprint: not peer reviewed.

[32] Wedeen VJ, Hagmann P, Tseng W-YI, Reese TG, Weisskoff RM. 2005. Mapping complex tissue architecture with diffusion spectrum magnetic resonance imaging. Magn Reson Med. 54:1377–1386. 10.1002/mrm.20642 16247738

[33] Zhang H, Schneider T, Wheeler-Kingshott CA, Alexander DC. 2012. NODDI: practical *in vivo* neurite orientation dispersion and density imaging of the human brain. Neuroimage. 61:1000–1016. 10.1016/j.neuroimage.2012.03.072 22484410

[34] Novikov DS, Fieremans E, Jespersen SN, Kiselev VG. 2019. Quantifying brain microstructure with diffusion MRI: theory and parameter estimation. NMR Biomed. 32:e3998. 10.1002/nbm.3998 PMC6481929 30321478

[35] Palombo M, et al 2020. SANDI: a compartment-based model for non-invasive apparent soma and neurite imaging by diffusion MRI. Neuroimage. 215:116835. 10.1016/j.neuroimage.2020.116835 PMC8543044 32289460

[36] Jelescu IO, de Skowronski A, Geffroy F, Palombo M, Novikov DS. 2022. Neurite exchange imaging (NEXI): a minimal model of diffusion in gray matter with inter-compartment water exchange. Neuroimage. 256:119277. 10.1016/j.neuroimage.2022.119277 PMC10363376 35523369

[37] Ramadan S . 2009. Diffusion-exchange weighted imaging. Magn Reson Insights. 3:MRI.S3504.

[38] Åslund I, Nowacka A, Nilsson M, Topgaard D. 2009. Filter-exchange PGSE NMR determination of cell membrane permeability. J Magn Reson. 200:291–295. 10.1016/j.jmr.2009.07.015 19647458

[39] Jelescu IO, et al 2025. Considerations and recommendations from the ISMRM diffusion study group for preclinical diffusion MRI: Part 1: in vivo small-animal imaging. Magn Reson Med. 93:2507–2534. 10.1002/mrm.30429 PMC11971505 40008568

[40] Schilling KG, et al 2025. Considerations and recommendations from the ISMRM diffusion study group for preclinical diffusion MRI: Part 2–Ex vivo imaging: added value and acquisition. Magn Reson Med. 93:2535–2560. 10.1002/mrm.30435 PMC11971501 40035293

[41] Callaghan PT, MacGowan D, Packer KJ, Zelaya FO. 1990. High-resolution *q*-space imaging in porous structures. J Magn Reson. 90:177–182.

[42] Karlicek RF, Lowe IJ. 1980. A modified pulsed gradient technique for measuring diffusion in the presence of large background gradients. J Magn Reson. 37:75–91.

[43] Özarslan E, Westin C-F, Mareci TH. 2015. Characterizing magnetic resonance signal decay due to Gaussian diffusion: the path integral approach and a convenient computational method. Concepts Magn Reson A. 44:203–213. 10.1002/cmr.a.21354 PMC4864615 27182208

[44] Barrick TR, Ingo C, Hall MG, Howe FA. 2025. Quasi-diffusion imaging: application to ultra-high b-value and time-dependent diffusion images of brain tissue. NMR Biomed. 38:e70011. 10.1002/nbm.70011 PMC11868825 40017343

[45] Hinrichs J, Kessler H. 1997. Fat content of milk and cream and effects on fat globule stability. J Food Sci. 62:992–995.

[46] Fieremans E, Lee H-H. 2018. Physical and numerical phantoms for the validation of brain microstructural MRI: a cookbook. Neuroimage. 182:39–61. 10.1016/j.neuroimage.2018.06.046 PMC6175674 29920376

