# Supplementary material for: Detecting and characterizing restricted diffusion with accessible magnetic field gradients
Source: PNAS Nexus. 2026 Sep 5;5(9):pgag293. doi: 10.1093/pnasnexus/pgag293 (PMC13586765; doi:10.1093/pnasnexus/pgag293)
Supplement: Supplementary file 1 [file pgag293_supplementary_data.pdf]

# Detecting and characterizing restricted diffusion with accessible magnetic field gradients – Supplementary Material

Alfredo Ordinola[1], Magnus Herberthson[2], Walker Scott Jackson[3], and Evren Özarslan[1,4]

[1] *Department of Biomedical Engineering, Linköping University, S-58185, Östergötland, Sweden*
[2] *Department of Mathematics, Linköping University, S-58330, Östergötland, Sweden*
[3] *Department of Biomedical and Clinical Sciences (BKV), Linköping University, S-58185, Östergötland, Sweden*
[4] *Center for Medical Image Science and Visualization (CMIV), Linköping University, Linköping, S-58185, Sweden*

## Free and restricted diffusion responses of gradient waveforms

In order to demonstrate the benefits of rPROP in the context of the experiments presented in Sec. 2 of the main article, we compare it to different gradient waveforms in Fig. S1a. These sequences are the traditional Stejskal-Tanner (ST) waveform; double pulsed field gradient (dPFG) waveform; pore space imaging (PSI) waveform. Furthermore, a special case of the dPFG waveform, the filter-probe double diffusion encoding (FP-DDE) is also analyzed. The waveforms are compared in two respects: (i) the the efficiency of free-diffusion filtering of the waveform, and (ii) its signal response for a fully restricted system (slab) in the long diffusion time regime.

As shown in Fig. S1b, for comparable sequence parameters (equal gradient pulse widths and maximum $q$-values), rPROP exhibits the narrowest signal response in a free-diffusion system among all compared waveforms, followed by PSI, dPFG, and ST, in that order. This shows that rPROP is the most efficient free-diffusion filter, i.e., it yields the smallest signal for the same $q$-value. Regarding the signal response in a restricted diffusion system, we observe that PSI exhibits the widest signal response, followed by rPROP and ST, and then by dPFG. As far as model fitting is concerned, as presented in Sec. 2.1 of the main article, it is beneficial to employ data which features a low free diffusion signal and large restricted diffusion signal, so that any modulation introduced by the system's geometry is accessible at low $q$-values and not heavily affected by noise. Considering this criterion and the results shown here, both rPROP and PSI could be deemed the best options for such studies, depending on hardware limitations and data quality. However, in this study, where achieving reliable measurements at low gradient amplitudes is our main objective, rPROP is identified as the most suitable waveform.

Comparisons with FP-DDE are less straightforward but lead to a similar conclusion. Note that in Fig.S1 we plotted the signal normalized by dividing it with the signal simulated with $q_f = q = 0$. If we had plotted the signal normalized by the signal obtained with $q = 0$ only, all FP-DDE curves would be identical to the ST one. And thus, its filtering performance is identical to ST. An additional limitation of FP-DDE concerns the substantial attenuation of the restricted-diffusion signal (at high $q_f$ values), producing data with reduced SNR.

Furthermore, the signal definition corresponding to the different waveforms in restricted diffusion is important to consider in order to appropriately compare them. In summary, for ideal scenarios (long diffusion time regime), PSI recovers the pore space function ($\rho(q)$) which is a static picture of the pore shape; while dPFG yields the same function for symmetric pores raised to the power of four ($|\rho(q)|^4$). In contrast, rPROP and ST yield the same response for ideal scenarios in restricted diffusion ($|\rho(q)|^2$). Because of this, we choose to compare the distributions reconstructed with ST and rPROP, namely the Ensemble Average Propagator (EAP) and the Mean Position Distribution (MPD), respectively, which hold dynamic information of the probed system. It should be stressed that these distributions are identical only for ideal scenarios in restricted diffusion, in any other settings they will deviate from one another, as shown in the results presented in Sec. 2.1 and 2.3 of the main article.

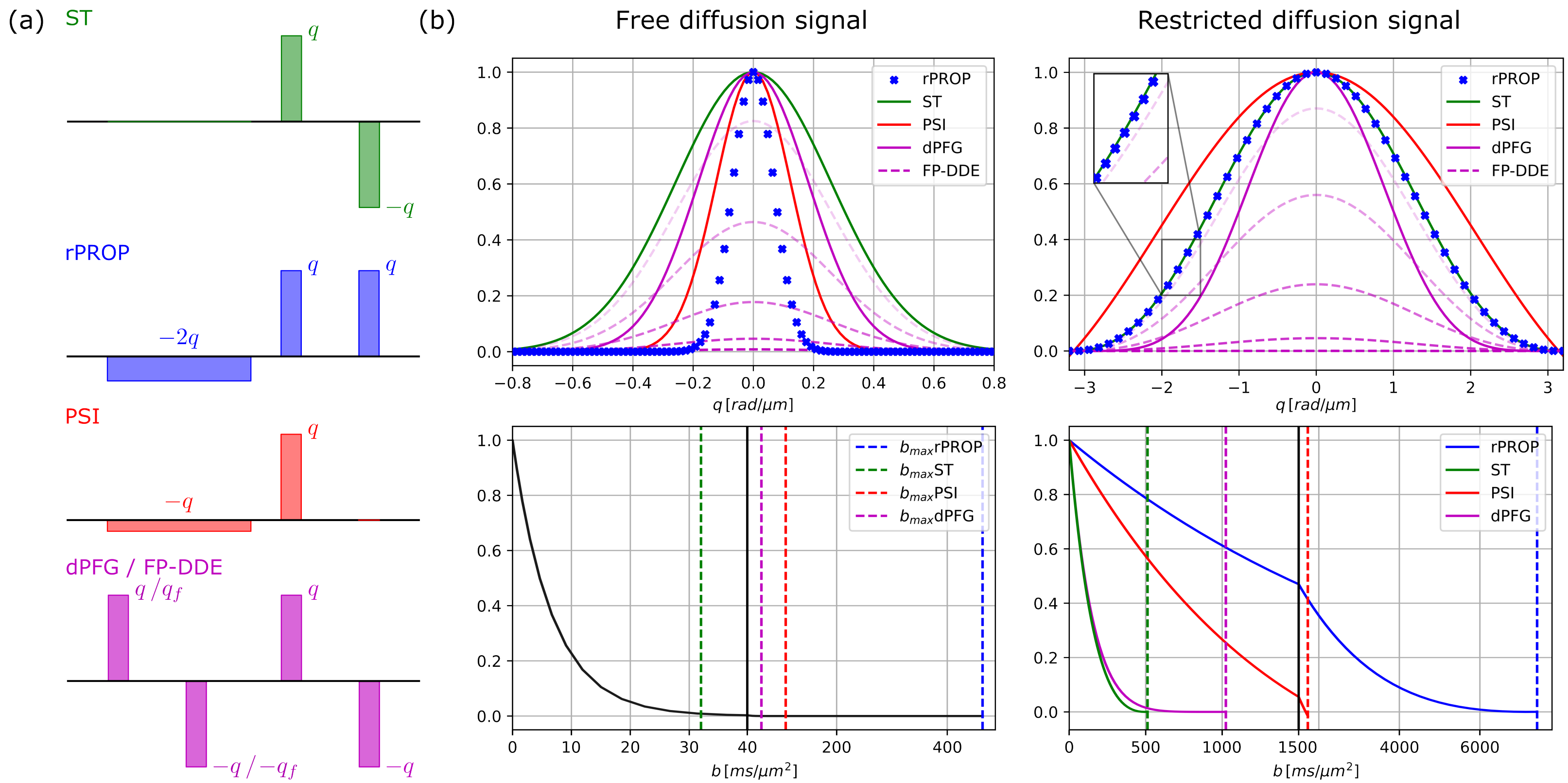


Figure S1: (a) Different diffusion gradient waveforms parametrized by a single gradient dependent term $q$. From top to bottom: the traditional Stejskal-Tanner (ST) waveform; the gradient waveform proposed in this study, reduced PROP (rPROP); pore space imaging (PSI) waveform; double pulsed field gradient (dPFG) waveform. A fifth waveform, referred to as filter-probe double diffusion encoding (FP-DDE), is defined by setting constant $q$-values in the first diffusion encoding of a dPFG waveform ($q_f$). (b) Free and restricted diffusion signals for all five sequences shown in (a) plotted with respect to $q$-value (top) and $b$-value (bottom). Different values of $q_f$ for FP-DDE are shown via dashed lines (the more transparent, the smaller $q_f$). In the signal vs. $b$-value plots, the solid black line corresponds to the free diffusion signal decay (which is the same for rPROP, ST, PSI, and dPFG), the dashed vertical lines correspond to the maximum diffusion weighting obtained with each waveform, and the solid vertical line corresponds to a change in axis spacing. Note that rPROP exhibits the narrowest signal response in the free diffusion system, showing that, for comparable sequence parameters, rPROP features the most effective free diffusion filter. The restricted diffusion response indicate that for an ideal scenario (long time regime) the signal for both ST and rPROP are equivalent (as shown in the zoomed in panel), while PSI and dPFG show a bigger and smaller signal magnitude, respectively, due to their signal definition in restricted systems. The FPE-DDE signals (dashed magenta) are plotted for decreasing values of $q_f$ and show good suppression of free diffusion for large filter values, however the same occurs to the restricted diffusion signal. The free diffusion system was defined with an intrinsic diffusivity ($D_f$) of $0.15\mu\text{m}^2/\text{ms}$ and the system with restricted diffusion in-between slabs had an intrinsic diffusivity ($D_0$) of $0.15\mu\text{m}^2/\text{ms}$ and length of $2\mu\text{m}$. Simulation parameters were narrow pulse width $\delta \to 0$, diffusion time $\Delta = 50\text{ms}$ for all gradient waveforms; and $\delta_0 = 500\text{ms}$ for PSI and rPROP. The $q_{\max}$ employed was $0.8\text{rad}.\mu\text{m}^{-1}$ for free diffusion and $3.2\text{rad}.\mu\text{m}^{-1}$ for restricted diffusion for all gradient waveforms.

# Additional analyses on the spinal cord sample

The signal values acquired from the spinal cord sample presented in the main article are also presented here. In Fig. S2, the signal averaged over voxels corresponding to WM and GM tissues is plotted in log-scale and against $q^2$ (bottom axis) and $b$-value (top axis). In such a plot, a truly a truly mono-exponential signal would lie on a line. This trend is observed neither in ST nor rPROP signals, however the deviation from mono-exponential decay is much more pronounced in rPROP than in ST, especially for gray matter (GM). Furthermore, the zoomed-in panels highlight the $q^2 < 750\text{mm}^{-2}$ range, for which the filter capabilities of rPROP are clearly observed. For ST within this range, we observe a linear decay of the signal and a small difference between GM and white matter (WM). On the contrary, for rPROP and the same range of $q^2$, we observe a considerable difference between each of the tissue's curves. Finally, from the $b$-value axes, we can clearly see how rPROP is capable of achieving much larger $b$-values than ST although the maximum gradient is the same.

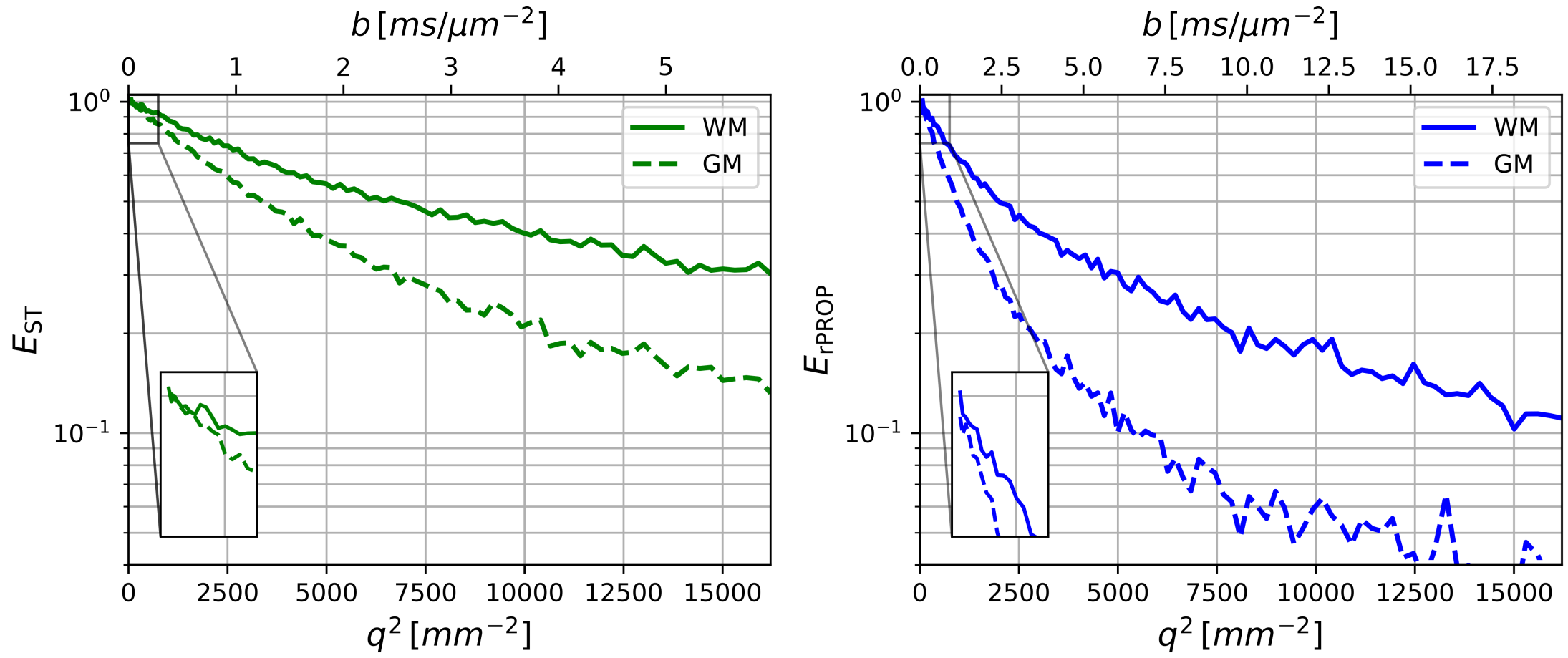


Figure S2: Signal values obtained by averaging data corresponding to white matter (WM) and gray matter (GM) of the spinal cord sample, plotted against $q^2$ (bottom axis) and $b$-value (top axis). To the left is the signal acquired with ST and to the right with rPROP. The zoomed in panel highlights the region of the signals with $q < 750\text{mm}^2$, where the filtering effect of rPROP is clearly observed.

In Fig. S3 we present a summary of the signals and distributions (ensemble average propagator – EAP – for ST and mean position distribution – MPD – for rPROP) acquired from WM and GM voxels in the spinal cord sample presented in the main article. Here, the solid and dashed lines correspond to mean values of all voxels and the width of their shading corresponds to their standard deviation. We observe that while standard deviation of the signals and distributions are fairly large, there is less overlap in the ones obtained with rPROP. Furthermore, in Fig. S4 we present signals and distributions from WM and GM of all three samples. Here we observe similar features as shown in the results from just SC281, suggesting differences between ST and rPROP are consistent across the different spinal cord samples studied in this work.
In order to review the reproducibility of the results shown in the spinal cord sample, data with the same protocol described in Sec. 6.3 of the main article was acquired for two additional spinal cord samples. The new samples were acquired as part of the same study mentioned in the main article and following national and local ethical guidelines (ethical permit number 14741-2019). Voxels corresponding to WM and GM for all samples were assigned using the Mean Diffusivity (MD) estimated via an independent DTI protocol to mitigate any inherent bias towards rPROP measurements. The EAP and MPD images for $\Delta x = 0$ and $\bar{x} = 0$ for each of the three samples are shown in Fig. S5, where we observe similar results in the two new samples (SC273 and SC279) as the ones reported for the spinal cord shown in the main article (SC281). That is, an improvement in tissue differentiation between white and gray matter using rPROP. The differences between WM and GM voxels computed with RTOP for ST and MPD at $\bar{x} = 0$ for rPROP were statistically significant ($p < 0.001$) for all three samples. It should be noted that values in the MPD maps exhibit a higher spread (standard deviation) in both tissues, which is mainly due to the voxels in the interface between WM and

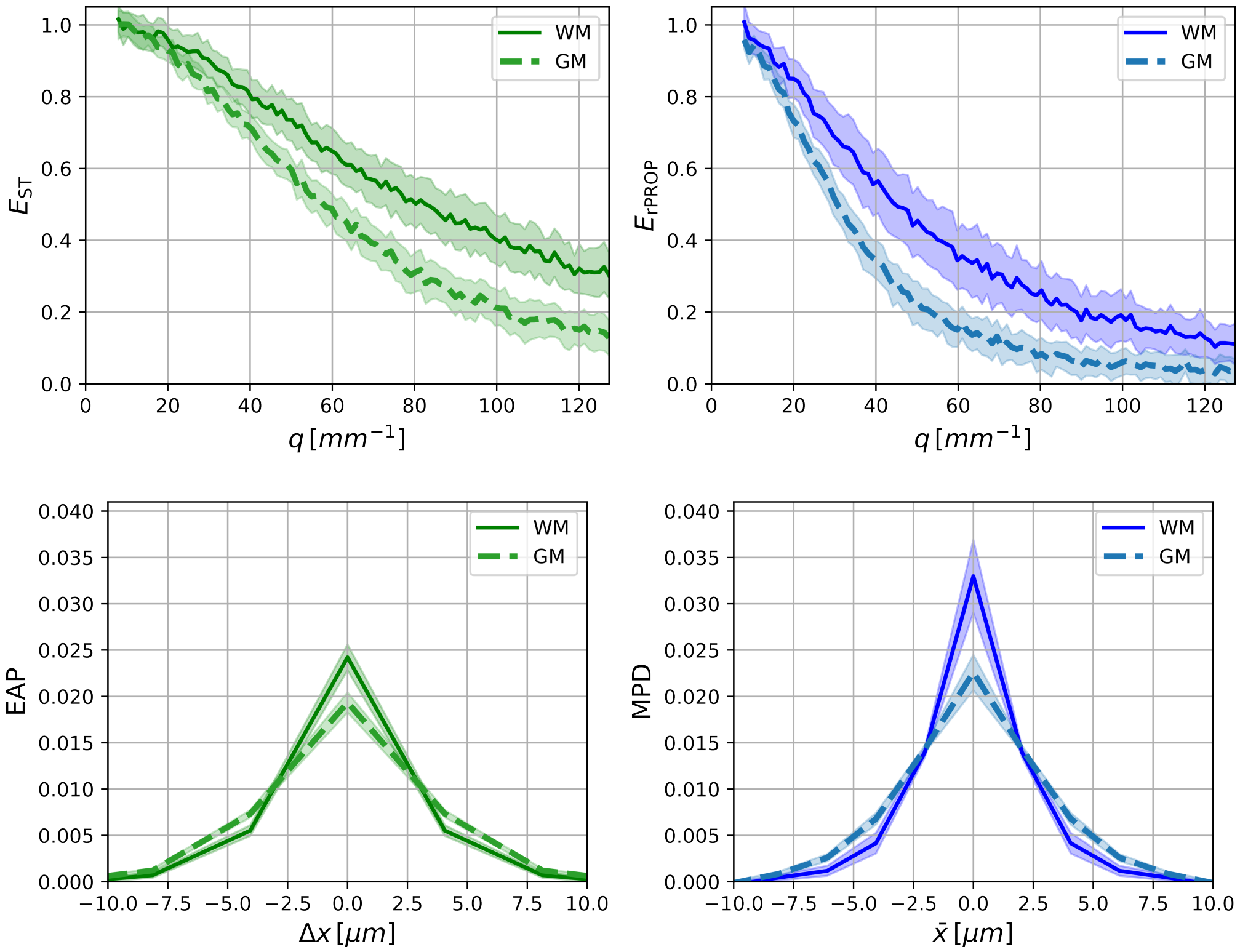


Figure S3: Signal values (top) and their inverse Fourier transforms (bottom) corresponding to a single sample: SC281. For ST data (left) this operation yields the ensemble average propagator (EAP) and for rPROP data (right), the mean position distribution (MPD). Solid lines correspond to the mean values of all WM voxels, and dashed lines to the mean values of all GM voxels. The width of the shading around the mean values correspond to twice the standard deviation ($\pm\sigma$) of signals and distributions.

GM, which were assigned to either tissue to compute the values shown in Fig. S5d.

Finally, to further showcase the benefits of rPROP, we calculated EAP and MPD images for $\Delta x = 0$ and $\bar{x} = 0$ for data with different maximum values of gradients ($g_{\max}$) for all three spinal cord samples. In other words, we truncated the signal from the right and only used data acquired up to a certain $g_{\max}$ to produce images akin to the ones in Fig. S5. The smallest value of $g_{\max}$ required for each method to achieve statistically significant differences ($p \leq 0.001$) between WM and GM are presented in Table S1. We obtained lower $g_{\max}$ values for rPROP across all three samples, even observing a reduction in gradient amplitude requirements as large as a factor of two (SC279), further demonstrating that rPROP can reduce the hardware requirements needed in tissue characterization studies.

Table S1: Maximum gradient amplitudes ($g_{\max}$) required to achieve statistically significant differences ($p \leq 0.001$) between WM and GM in spinal cord samples.

| Method | Quantity | SC273 | SC279 | SC281 |
|---|---|---|---|---|
| ST | EAP ($\Delta x = 0$) | 0.221T/m | 0.306T/m | 0.221T/m |
| rPROP | MPD ($\bar{x} = 0$) | 0.164T/m | 0.150T/m | 0.150T/m |

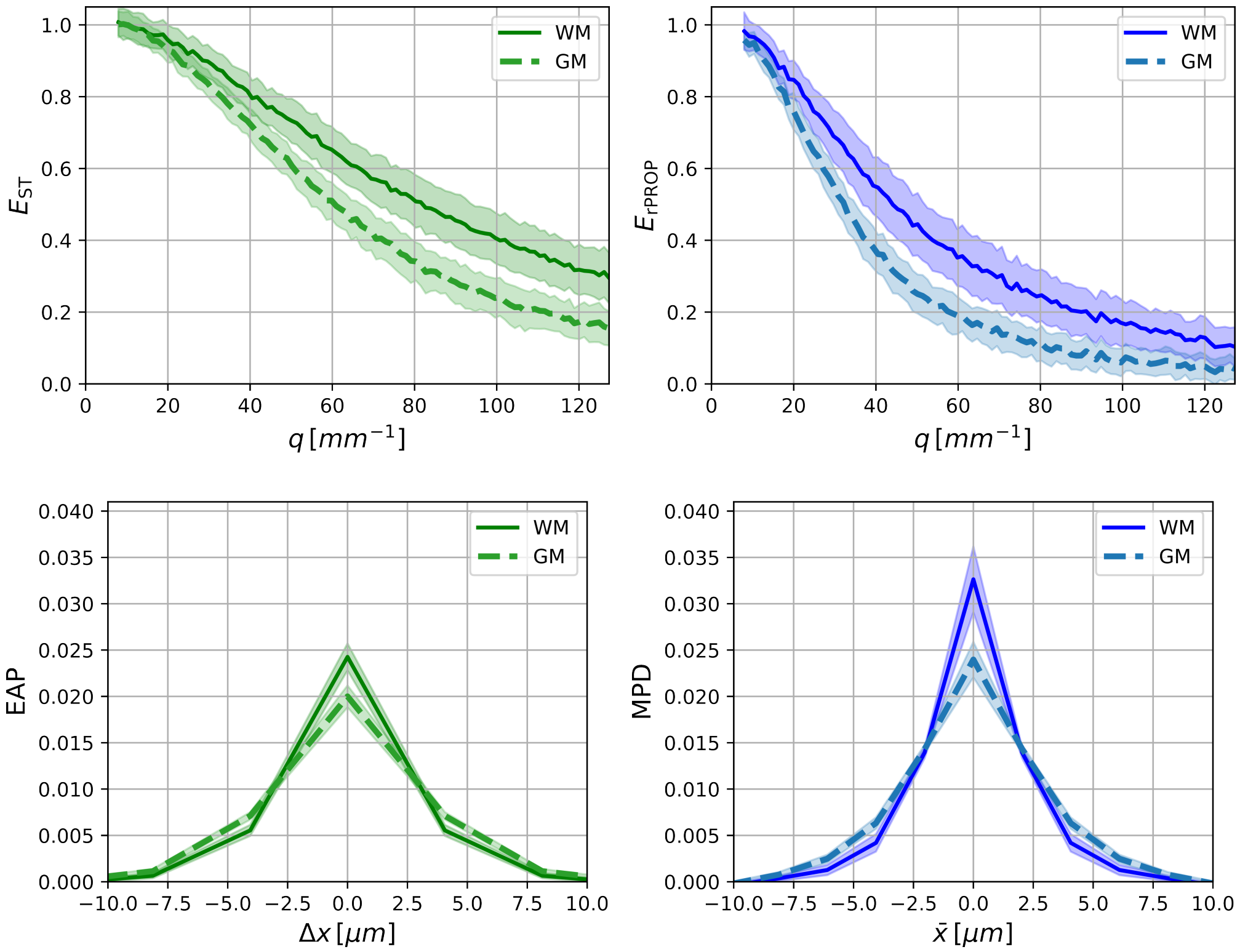


Figure S4: Signal values (top) and their inverse Fourier transforms (bottom) obtained from all three samples: SC273, SC279 and SC281. For ST data (left) this operation yields the ensemble average propagator (EAP) and for rPROP data (right), the mean position distribution (MPD). Solid lines correspond to the mean values of all WM voxels in all three samples, and dashed lines to the mean values of all GM voxels in all three samples. The width of the shading around the mean values correspond to twice the standard deviation ($\pm\sigma$) of signals and distributions.

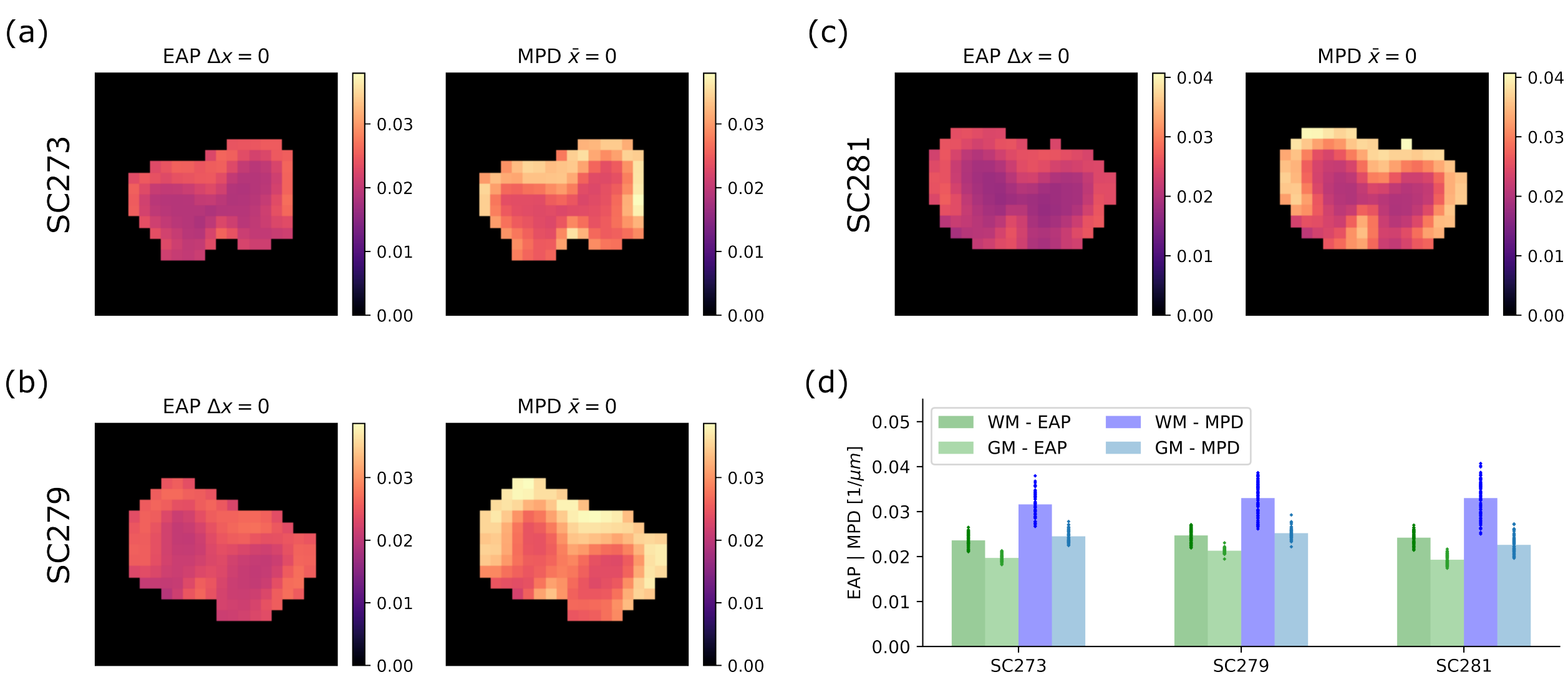


Figure S5: Estimated EAP and MPD images for $\Delta x = 0$ and $\bar{x} = 0$, respectively, for three different spinal cord samples: (a) SC273, (b) SC279, and (c) SC281 (sample shown in the main article). (d) Bar plot with mean values of the maps (a)-(c) of white matter (WM) and gray matter (GM) voxels. Scattered points show individual voxel values for each distribution map and tissue.

## Tensors accessible via rPROP in closed pores

At low $\boldsymbol{q}$-values, Eq. 3 of the main article can be approximated with the following expression:

$$\begin{aligned} E_{\mathrm{ST}}(\boldsymbol{q}) &\approx \int_\Omega \mathrm{d}\boldsymbol{R}\,\left[1 - i\boldsymbol{q}\cdot\boldsymbol{R} - (\boldsymbol{q}\cdot\boldsymbol{R})^2\right]\,\hat{p}_\Delta(\boldsymbol{R}) \\ &\approx \exp\left[-\int \mathrm{d}\boldsymbol{R}\,(\boldsymbol{q}\cdot\boldsymbol{R})^2\,\hat{p}_\Delta(\boldsymbol{R})\right] = \exp\left[-\boldsymbol{q}^{\mathrm{T}}\left\langle \boldsymbol{R}\,\boldsymbol{R}^{\mathrm{T}}\right\rangle \boldsymbol{q}\right] \\ &= \exp\left[-\left(q_x^2\langle\Delta x^2\rangle + q_y^2\langle\Delta y^2\rangle + q_z^2\langle\Delta z^2\rangle + 2q_xq_y\langle\Delta x\Delta y\rangle + 2q_xq_z\langle\Delta x\Delta z\rangle + 2q_yq_z\langle\Delta y\Delta z\rangle\right)\right]\ , \end{aligned} \tag{S1}$$

where $\Delta x$, $\Delta y$, and $\Delta z$ are the components of the net displacement vector ($\boldsymbol{R}$), and $q_x$, $q_y$, and $q_z$ are the components of the $\boldsymbol{q}$-vector. The six unique quantities inside the operator $\langle\ldots\rangle$ (which denotes the expected value) are then used to define a tensor, which we refer to as the mean squared displacement tensor. Note that a DTI analysis with $b$-matrix $b_{ij} \propto q_iq_j$ reveals the traditional diffusion tensor with its corresponding components $D_{i,j}$, and can be performed by rewriting Eq. S1 in the following manner:

$$E_{\mathrm{ST}}(\boldsymbol{b}) \approx \exp\left[-\left(b_{xx}D_{xx} + b_{yy}D_{yy} + b_{zz}D_{zz} + 2b_{xy}D_{xy} + 2b_{xz}D_{xz} + 2b_{yz}D_{yz}\right)\right]\ . \tag{S2}$$

At low $\boldsymbol{Q}$-values, Eq. 5 of the main article can be rewritten as

$$\begin{aligned} E_{\mathrm{rPROP}}(\boldsymbol{Q}) &\approx \int \mathrm{d}\bar{\boldsymbol{r}}\,\left[1 - i\boldsymbol{Q}\cdot\bar{\boldsymbol{r}} - (\boldsymbol{Q}\cdot\bar{\boldsymbol{r}})^2\right]\,\bar{p}_\Delta(\bar{\boldsymbol{r}}) \\ &\approx \exp\left[-\int \mathrm{d}\bar{\boldsymbol{r}}\,(\boldsymbol{Q}\cdot\bar{\boldsymbol{r}})^2\,\bar{p}_\Delta(\bar{\boldsymbol{r}})\right] = \exp\left[-\boldsymbol{Q}^{\mathrm{T}}\left\langle \bar{\boldsymbol{r}}\,\bar{\boldsymbol{r}}^{\mathrm{T}}\right\rangle \boldsymbol{Q}\right] \\ &= \exp\left[-\left(Q_x^2\langle\bar{x}^2\rangle + Q_y^2\langle\bar{y}^2\rangle + Q_z^2\langle\bar{z}^2\rangle + 2Q_xQ_y\langle\bar{x}\bar{y}\rangle + 2Q_xQ_z\langle\bar{x}\bar{z}\rangle + 2Q_yQ_z\langle\bar{y}\bar{z}\rangle\right)\right]\ , \end{aligned} \tag{S3}$$

where $\bar{x}$, $\bar{y}$, and $\bar{z}$ are the components of the mean position vector ($\bar{\boldsymbol{r}}$), and $Q_x$, $Q_y$, and $Q_z$ are the components of the $\boldsymbol{Q}$ vector. Similarly to the derivation above, the six unique components within the expected value operator are used to define a new tensor, which we refer to as the mean position tensor.
Recalling that $\boldsymbol{R} = (\boldsymbol{r'} - \boldsymbol{r})$ and $\bar{\boldsymbol{r}} = (\boldsymbol{r} + \boldsymbol{r'})/2$, the following 12 unique components can be obtained through simple algebraic manipulations using the quantities in Eqs. S1 and S3: $\langle x^2\rangle$, $\langle y^2\rangle$, $\langle z^2\rangle$, $\langle xy\rangle$, $\langle xz\rangle$, $\langle yz\rangle$, $\langle xx'\rangle$, $\langle yy'\rangle$, $\langle zz'\rangle$, $\langle xy'\rangle$, $\langle xz'\rangle$, $\langle yz'\rangle$. For example, given the components $\langle\bar{x}^2\rangle$ and $\langle\Delta x^2\rangle$ are estimated, they can be rewritten as:

$$\left\langle\bar{x}^2\right\rangle = \left\langle\left(\frac{x + x'}{2}\right)^2\right\rangle = \frac{\left\langle x^2\right\rangle}{4} + \frac{\langle xx'\rangle}{2} + \frac{\left\langle x'^2\right\rangle}{4} \tag{S4}$$

$$\left\langle\Delta x^2\right\rangle = \left\langle(x' - x)^2\right\rangle = \left\langle x'^2\right\rangle - 2\left\langle xx'\right\rangle + \left\langle x^2\right\rangle \tag{S5}$$

In closed pores, it follows that $\langle x^2\rangle = \langle x'^2\rangle$, which along with the following linear operations result in the $\langle x^2\rangle$ and $\langle xx'\rangle$ components:

$$\left\langle\bar{x}^2\right\rangle + \frac{\left\langle\Delta x^2\right\rangle}{4} = \frac{\left\langle x^2\right\rangle}{2} + \frac{\left\langle x^2\right\rangle}{2} = \left\langle x^2\right\rangle \tag{S6}$$

$$\left\langle\bar{x}^2\right\rangle - \frac{\left\langle\Delta x^2\right\rangle}{4} = \frac{\langle xx'\rangle}{2} + \frac{\langle xx'\rangle}{2} = \langle xx'\rangle \tag{S7}$$

The other 10 unique components can be similarly calculated, from which two new symmetric tensors can be defined. The first, with components $\langle x^2\rangle$, $\langle y^2\rangle$, $\langle z^2\rangle$, $\langle x\,y\rangle$, $\langle x\,z\rangle$, and $\langle y\,z\rangle$ describes the likelihood of particles' initial position to be correlated. The second, with components $\langle x\,x'\rangle$, $\langle y\,y'\rangle$, $\langle z\,z'\rangle$, $\langle x\,y'\rangle$, $\langle x\,z'\rangle$, and $\langle y\,z'\rangle$, describes correlations between the particles' initial and final positions. Note that by initial and final positions we refer to the positions encoded by the first and second narrower pulses, respectively. Additionally, from the first tensor, the moment of inertia of the particles in a given compartment along different directions can be calculated with the expression

$$\mathbf{I} = \left\langle\boldsymbol{r}^{\mathrm{T}}\,\boldsymbol{r}\right\rangle\mathbb{I} - \left\langle\boldsymbol{r}\,\boldsymbol{r}^{\mathrm{T}}\right\rangle\ , \tag{S8}$$

i.e.,

$$I_{xx} = \langle y^2 \rangle + \langle z^2 \rangle \tag{S9a}$$

$$I_{yy} = \langle x^2 \rangle + \langle z^2 \rangle \tag{S9b}$$

$$I_{zz} = \langle x^2 \rangle + \langle y^2 \rangle \tag{S9c}$$

$$I_{xy} = \langle I_{yx} \rangle = - \langle xy \rangle \tag{S9d}$$

$$I_{xz} = \langle I_{zx} \rangle = - \langle xz \rangle \tag{S9e}$$

$$I_{yz} = \langle I_{yz} \rangle = - \langle yz \rangle \ , \tag{S9f}$$

where $\mathbb{I}$ is the identity matrix, and $\mathbf{I}$ is the moment of inertia tensor assuming the total particle mass equals unity.
This tensor quantifies the resistance of the particles within a certain compartment against rotations about differently-oriented axes, which is closely related to the geometry of such a compartment. For example, in a cylinder-like compartment aligned along the $y$-axis, we expect low values in the $I_{yy}$ component of the moment of inertia tensor, as the molecules within this cylinder will offer little resistance to rotations along the $y$-axis, while the opposite is expected for the $I_{xx}$ and $I_{zz}$ components of this tensor.

# Direction distribution in multidimensional analyses

The set of directions probed in the multidimensional analysis (arranged based on tessellation of an icosahedron on the hemisphere) in Sec. 2.2 of the main article is presented in Table S2. Note that only the first 6 directions were employed for lower gradient amplitudes, while all 21 directions were used for higher gradient amplitudes, yielding a total of 27 signals per dataset.

Table S2: Unit vectors of the direction set in the multidimensional analyses. The first 6 directions were employed for lower gradient amplitudes, while all 21 directions were used for higher gradient amplitudes.

| $(x, y, z)$ |
|---|
| (0,0,1) |
| (0.8944,0,0.4472) |
| (0.2764,0.8507,0.4472) |
| (-0.7236,0.8507,0.4472) |
| (-0.7236,-0.8507,0.4472) |
| (0.2764,-0.8507,0.4472) |
| (0.5257,0,0.8507) |
| (0.1626,0.5,0.8507) |
| (-0.4253,0.3090,0.8507) |
| (-0.4253,-0.309,0.8507) |
| (0.1625,-0.5,0.8507) |
| (0.6882,0.5,0.5257) |
| (0.6882,-0.5,0.5257) |
| (0.9511,0.309,0) |
| (0.9511,-0.309,0) |
| (-0.2696,0.809,0.5257) |
| (0.5878,0.809,0) |
| (0,1,0) |
| (-0.8507,0,0.5257) |
| (-0.2629,-0.809,0.5257) |
| (0.5878,-0.809,0) |
